# Homologous recombination deficiency status-based classification of high-grade serous ovarian carcinoma

**DOI:** 10.1038/s41598-020-59671-3

**Published:** 2020-02-17

**Authors:** Hisamitsu Takaya, Hidekatsu Nakai, Shiro Takamatsu, Masaki Mandai, Noriomi Matsumura

**Affiliations:** 1 Kindai University Faculty of Medicine, Department of Obstetrics and Gynecology, Osakasayama, 589-8511 Japan; 2 Kyoto University Graduate School of Medicine, Department of Gynecology and Obstetrics, Kyoto, 606-8507 Japan

**Keywords:** Cancer genomics, Ovarian cancer

## Abstract

Homologous recombination repair (HRR) pathway deficiency (HRD) is involved in the tumorigenesis and progression of high-grade serous ovarian carcinoma (HGSOC) as well as in the sensitivity to platinum chemotherapy drugs. In this study, we obtained data from The Cancer Genome Atlas (TCGA) on HGSOC and identified scores for the loss of heterozygosity, telomeric allelic imbalance, and large-scale state transitions, and calculated the HRD score. We then investigated the relationships among the score, genetic/epigenetic alterations in HRR-related genes, and the clinical data. We found that *BRCA1/2* mutations were enriched in the group with HRD scores ≥63. Compared with the groups with scores ≤62, this group had a good prognosis; we thus considered HRD scores ≥63 to be the best cutoff point for identifying HRD cases in HGSOC. Classification of HGSOC cases by the HRD status revealed a better prognosis for HRD cases caused by genetic alterations (genetic HRD) than those caused by epigenetic changes and those caused by undetermined reasons (p = 0.0002). Among cases without macroscopic residual tumors after primary debulking surgery, 11 of 12 genetic HRD cases survived after the median observation period of 6.6 years, showing remarkably high survival rates (p = 0.0059). In conclusion, HGSOC can be classified into subtypes with different prognoses according to HRD status. This classification could be useful for personalized HGSOC treatment.

## Introduction

Ovarian cancer has the worst prognosis of all gynecologic malignancies^1^. Specifically, high-grade serous ovarian cancer (HGSOC), which is the most common type of ovarian cancer, is mostly advanced stage III–IV disease at the time of diagnosis^2^. The primary treatment approach for this cancer is a combination of debulking surgery and a platinum-chemotherapy. However, the majority of ovarian cancer patients experience tumor recurrence; they develop chemotherapy resistance that ultimately becomes lethal^3^.

HGSOC is characterized by chromosomal instability due to homologous recombination repair (HRR) pathway deficiency (HRD)^4^. Germline *BRCA1/2* mutations, somatic *BRCA1/2* mutations, and *BRCA* gene promotor methylations are well-known causes of HRD, but other genetic abnormalities of the HRR pathway could also cause HRD^4,5^, although no consensus has been reached. The presence of HRD results in irreparable DNA damage from platinum-containing drugs, which leads to cell death. Moreover, an underlying HRD in tumor cells makes the cells sensitive to PARP inhibitors. PARP inhibitors bind to and trap PARP1 and PARP2 on DNA at the sites of single-strand breaks, which results in the generation of double-strand breaks. In cancer cells with HRD, double-strand DNA breaks are repaired by error-prone pathways (i.e. nonhomologous end joining), ultimately leading to cell death^6^.

HRD causes characteristic genomic scar signatures, namely, the loss of heterozygosity (LOH)^7^, telomeric allelic imbalance (TAI)^8^, and large-scale state transitions (LST)^9^. The HRD score is the sum of these scar signature scores^10^. The HRD score correlates with sensitivity to niraparib, which is a PARP inhibitor^11^.

In The Cancer Genome Atlas (TCGA) project, about half of HGSOC cases are reported to have HRD due to an HRR pathway abnormality^4^. However, the relationship between the HRD score and HRR pathway gene abnormalities other than those in *BRCA1/2* has not been thoroughly investigated. We thus investigated the connection between HRD status and HRR pathway gene abnormalities in HGSOC data in the TCGA, and we show that HGSOC can be classified according to HRD status into subtypes with different prognoses.

## Results

**Figure 1:**
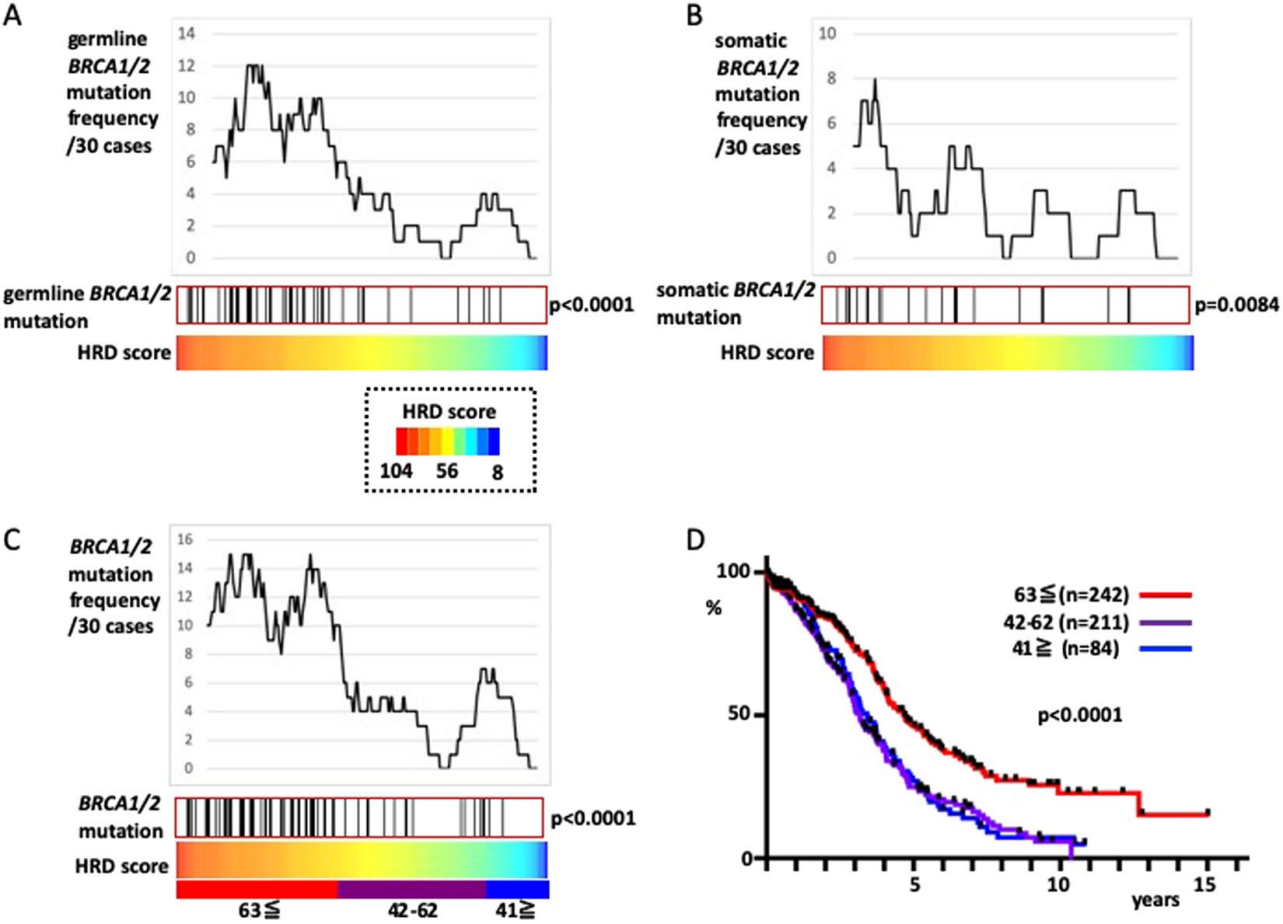
Definition of HRD cases according to HRD scores. The 296 cases for which both exome sequence and SNP array data were available were sorted by HRD score. (**A**) Distribution of cases with germline *BRCA1/2* mutations. The upper graph shows the frequency of germline *BRCA1/2* mutation cases by sliding the windows of 30 cases. The black bars in the central box represent germline *BRCA1/2* mutations cases. (**B**) Distribution of cases with somatic *BRCA1/2* mutations. (**C**) germline *BRCA1/2* mutations and somatic *BRCA1/2* mutations are presented together as *BRCA1/2* mutations; these cases are concentrated in HRD scores ≥63. The cutoff HRD score ≥42, which is generally used, is also shown. D) Comparison of overall survival rate by HRD scores; 537 cases with known prognosis and SNP array data were divided according to HRD scores ≥63, 42–62, and ≥41.

First, we defined HRD cases by investigating the distribution of cases that harbored germline *BRCA1/2* or somatic *BRCA1/2* mutations. Compared with the cases without germline *BRCA1/2* mutations, those cases with germline *BRCA1/2* mutations had higher scores for LOH^7^ (p = 0.0018), TAI^8^ (p < 0.0001), and LST^9^ (p < 0.0001). Cases with somatic *BRCA1/2* mutations scored higher for LOH compared with those without somatic *BRCA1/2* mutations (p = 0.0018; p = 0.28 and 0.06 for TAI and LST scores, respectively). HRD scores (TAI + LST + LOH) were higher in those cases with germline *BRCA1/2* mutations or somatic *BRCA1/2* mutations, compared with those without (Fig. 1A,B; p = 0.0001 and 0.0084 for germline *BRCA1/2* mutations and somatic *BRCA1/2* mutations, respectively). When germline *BRCA1/2* mutations and somatic *BRCA1/2* mutations were analyzed together as *BRCA* mutations, it was found that *BRCA* mutation cases had high HRD scores (p < 0.0001, Fig. 1C).

Until now, the proposed cutoff value for the HRD score has been ≥42^10^. In the report where this cutoff was proposed, the ovarian cancer and breast cancer datasets were analyzed together. Therefore, we also analyzed the breast cancer TCGA dataset^12^, and found that generally, breast cancer had lower HRD scores than HGSOC (Supplementary Fig. 1A, p < 0.0001), but higher HRD scores were still associated with *BRCA* mutation cases (p < 0.0001, Supplementary Fig. 1B). Combined analysis of ovarian and breast cancer datasets from 1257 cases showed that the distribution of *BRCA* mutation cases was bimodal. Indeed, there also seemed to be a cutoff at HRD scores ≥42 (Supplementary Fig. 1C). However, in the analysis of ovarian cancer alone, *BRCA* mutation cases were more likely to have HRD scores ≥63 (Fig. 1C). *BRCA* mutations represented 38% of the cases with HRD scores ≥63 (49/128), 10% with HRD scores between 42 and 62 (12/118), and 10% with HRD scores 41 and below (5/50); no enrichment of *BRCA* mutation cases with HRD scores from 42 to 62 was observed compared with those with scores ≤41. An analysis of the prognosis revealed overlap in the survival curves of patients with HRD scores of 42–62 (n = 211) and ≤41 (n = 84), but the survival rate of patients with HRD scores ≥63 (n = 242) was clearly better than that of the other two groups (Fig. 1D, p < 0.0001). Thus, we designated an HRD score ≥63 as the cutoff.

**Figure 2:**
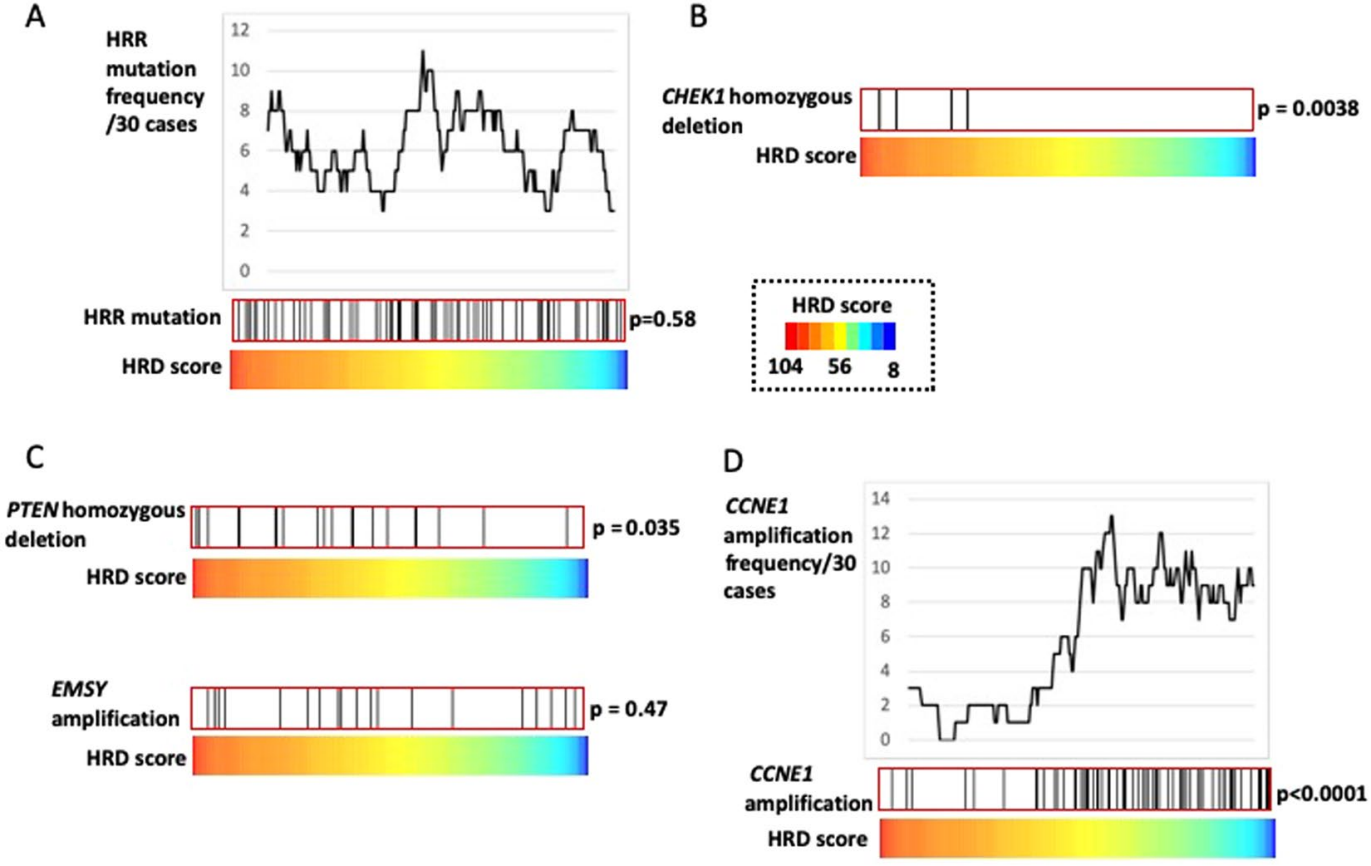
Relationship between HRD scores and mutations in HRR-related genes other than *BRCA1/2*. Dividing 296 cases according to HRD scores shows the distribution of cases with mutations. (**A**) Distribution of cases with HRR pathway gene mutations other than those in *BRCA1/2*, including homozygous deletions. (**B**) *CHEK1* homozygous deletions had high HRD scores among the HRR pathway gene mutations other than those in *BRCA1/2*. (**C**) *PTEN* homozygous deletions and *EMSY* amplifications are two gene mutations that may cause HRD. (**D**) Relationship between *CCNE1* amplifications and HRD score; *CCNE1* amplification cases were enriched in low HRD scores.

Subsequently, we investigated the connection between the HRD score and overall HRR pathway gene mutations other than *BRCA* mutations but found none (Fig. 2A, p = 0.58). Moreover, no difference was observed in survival rates between patients with and without these HRR mutations (p = 0.78, data not shown). However, individual analyses of these HRR mutations revealed that the *CHEK1* homozygous deletions was associated with high HRD scores (Fig. 2B, Supplementary Fig. 2, p = 0.0038). The *PTEN* homozygous deletions and *EMSY* amplifications are candidate mutations for causing HRD^5^. Of these, *PTEN* homozygous deletions were positively correlated with HRD scores (p = 0.035), whereas *EMSY* amplifications were not (Fig. 2C, Supplementary Fig. 2). *CCNE1* amplifications, a genetic abnormality that characterizes HR-proficient HGSOC^5^, were clearly associated with low HRD scores (Fig. 2D; p < 0.0001).

**Figure 3:**
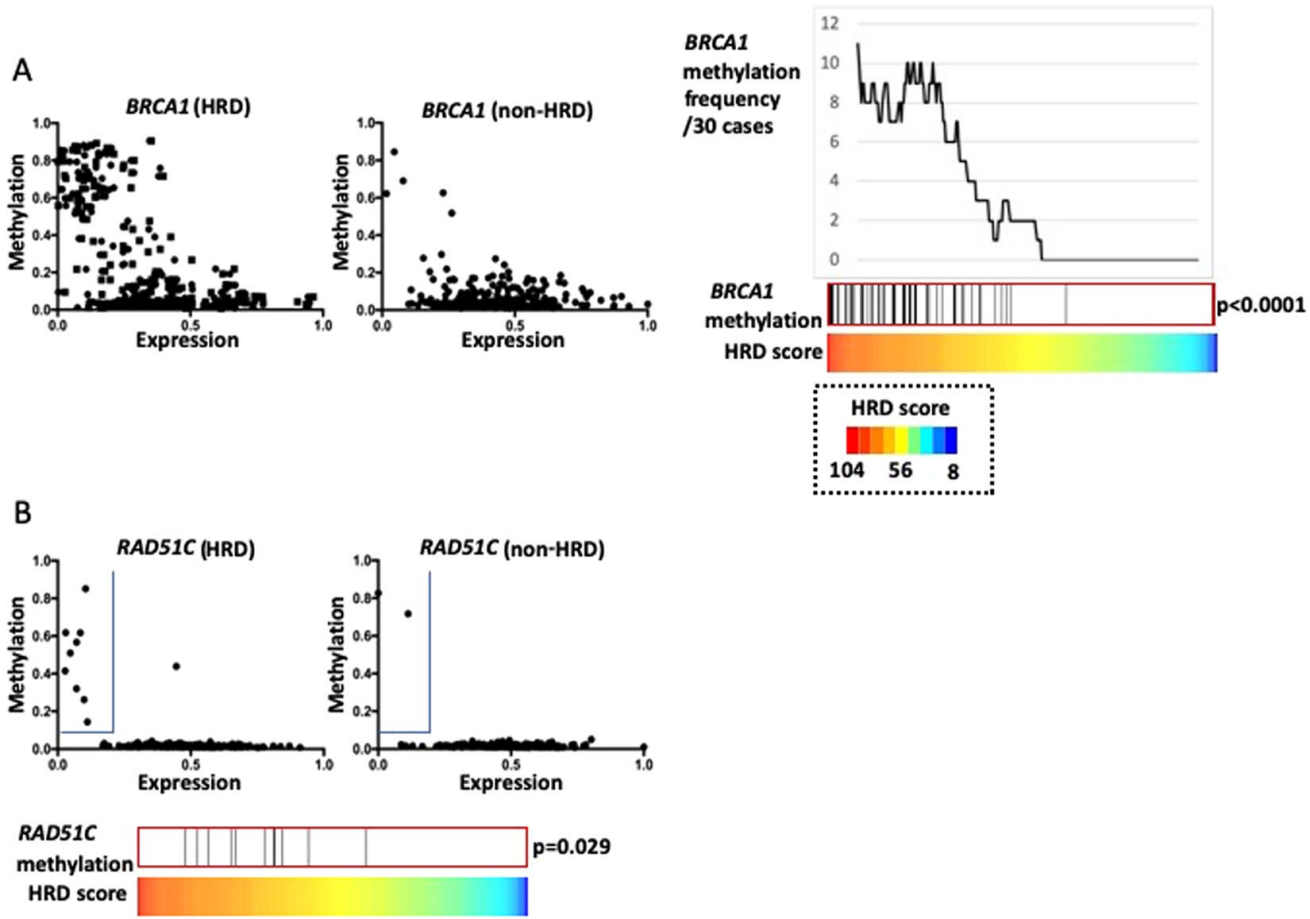
Relationship between HRD status and DNA promoter methylation of HRR-related genes. (**A**) *BRCA1* methylation. Left: HRD cases; Center: non-HRD cases. X-axis: Average values of two *BRCA1* mRNA expression probes normalized to 0–1. Y-axis: β value of cg04658354. Right: Distribution of *BRCA1* methylated cases. (**B**) *RAD51C* methylation. X-axis: Average values of two *RAD51C* mRNA expression probes normalized to 0–1. Y-axis: β value of cg14837411. *RAD51C* methylation cases are surrounded by a blue line in the upper-left region. The frequency of *RAD51C* methylation differs greatly between HRD cases (left) and non-HRD cases (right).

Next, we analyzed DNA methylation in the promoter region of HRR-related genes. First, we investigated *BRCA1* methylation. Of the nine *BRCA1* methylation probes, the β value of cg04658354 was most negatively correlated with the *BRCA1* mRNA expression level (r = −0.49). When *BRCA1* mRNA expression and cg04658354 β values were plotted, markedly more methylated cases with a high β value and low mRNA expression were observed in the HRD group than in the non-HRD group (Fig. 3A). The 56 *BRCA1* methylated cases described in the TCGA paper^4^ revealed higher HRD scores than non-methylated cases (Fig. 3A, p < 0.0001). Similarly, *RAD51C* methylation probe cg14837411 showed strong negative correlation with the mRNA expressions (r = −0.40). Cases with methylation, which were defined by a β value > 0.1 and a normalized expression <0.2, were concentrated in the high HRD cases (p = 0.029, Fig. 3B). Among the other HRR pathway-related genes, the only one with a strong negative correlation between the methylation probe β value and gene expression was *PTEN* cg21573601 (r = −0.40). However, the maximum β value of this probe was low (0.12), and it is thus unclear whether this gene was actually methylated. Candidate *PTEN* methylation cases selected using a cutoff β value > 0.03 and a normalized expression <0.4 had high HRD scores (p = 0.0022, Supplementary Fig. 3).

**Figure 4:**
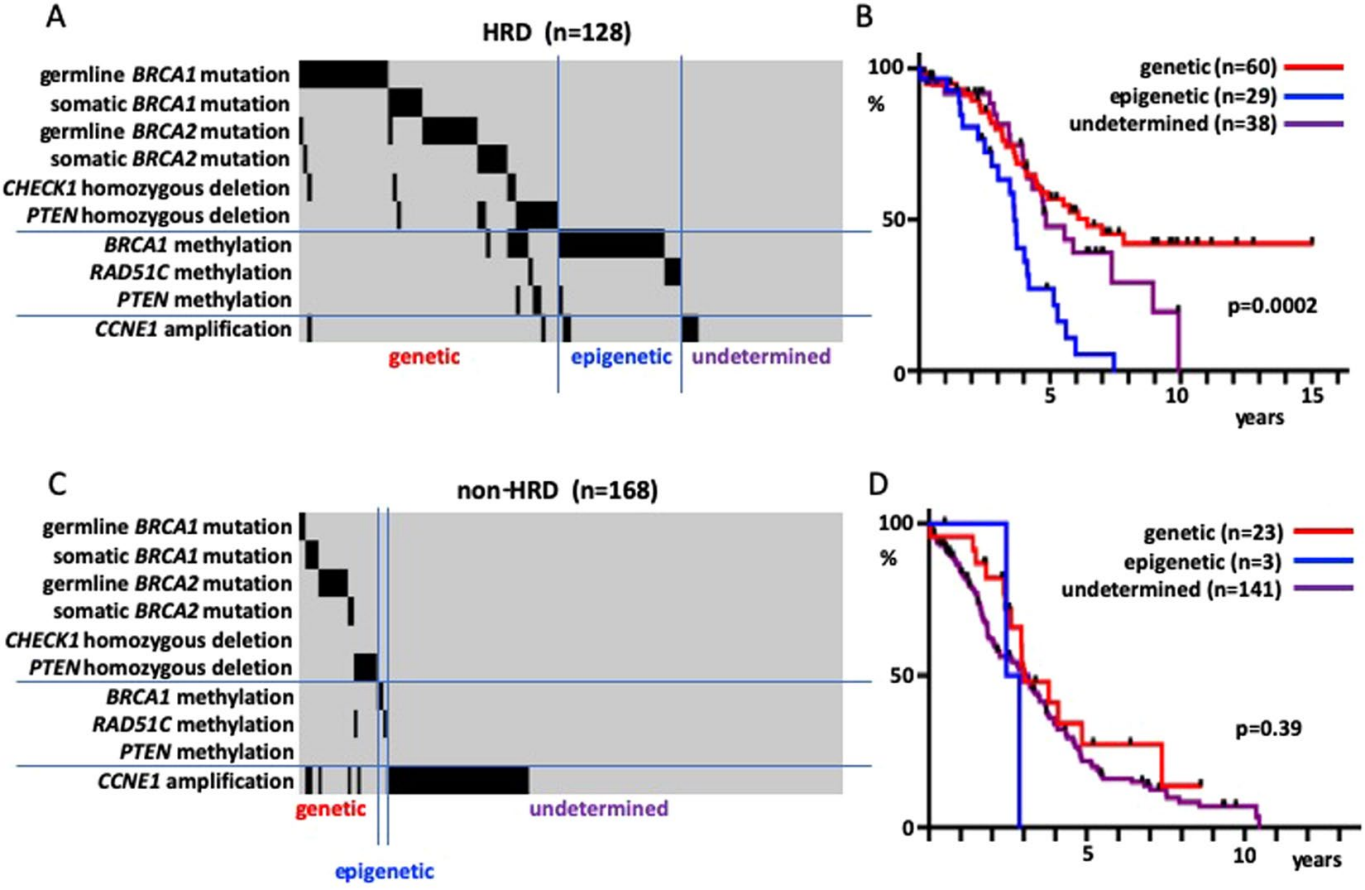
Relationship between the molecular mechanism of HRD and prognosis; sorting of cases by mechanism into the genetic mutation (genetic), DNA methylation (epigenetic), and unknown mechanism (undetermined) groups. (**A**) Classification of HRD cases. (**B**) Survival rate of HRD cases. (**C**) Classification of non-HRD cases. (**D**) Survival rate of non-HRD cases.

Considering the above findings, the HRD cases for which both SNP array and exome sequence data were available (n = 128) were classified according to the underlying molecular mechanism. Those cases that had one of the six factors that could induce HRD genetically (germline *BRCA1* mutations, somatic *BRCA1* mutations, germline *BRCA2* mutations, somatic *BRCA2* mutations, *CHEK1* homozygous deletions, and *PTEN* homozygous deletions) were classified as genetic HRD. Other cases that had *BRCA1* methylations or *RAD51C* methylations were classified as epigenetic HRD, while the remainder were classified as undetermined HRD. There was no case with *PTEN* candidate methylation alone (Fig. 4A). The survival rates of these three groups (comprising 127 patients for which the overall survival (OS) data were available) revealed a good prognosis for genetic HRD and a poor prognosis for epigenetic HRD (Fig. 4B, p = 0.0002). No difference was found in the prognosis between genetic HRD cases with *BRCA1/2* mutations and those with *CHEK1* homozygous deletions or *PTEN* homozygous deletions. Since there were only a few *RAD51C* methylation cases among the epigenetic HRD cases, it was difficult to compare their prognoses with those of the *BRCA1* methylation cases (Supplementary Fig. 4A). A similar classification of the non-HRD cases (n = 168; n = 167 for those with OS information; Fig. 4C) revealed no difference in survival rates (Fig. 4D, p = 0.39). Additionally, a survival rate comparison between HRD and non-HRD cases with the aforementioned six factors causing genetic HRD revealed a better prognosis for HRD cases (Supplementary Fig. 4B, p = 0.019).

**Figure 5:**
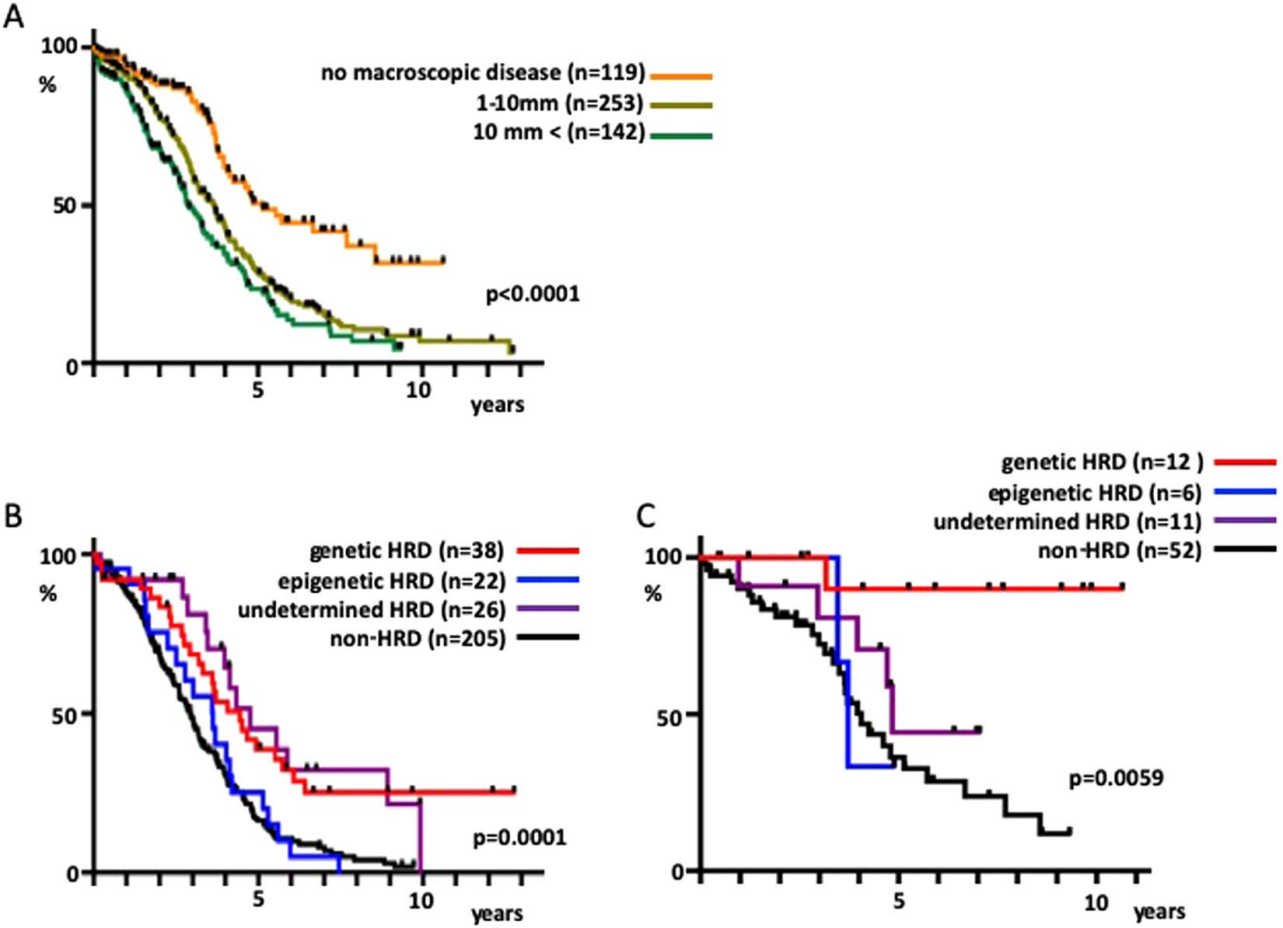
Relationship among residual tumor at the time of surgery, HRD status, and prognosis. (**A**) The 514 cases were sorted by size of the residual tumor if data on residual tumor at the time of surgery were available (no macroscopic disease, 1–10 mm and >10 mm) and their survival rates were compared. (**B**) Cases with residual tumor ≥1 mm and available SNP array data were divided into the HRD and non-HRD groups. HRD cases with exome sequencing data were further sorted by molecular mechanism into genetic HRD, epigenetic HRD, and undetermined HRD to compare the survival rate. (**C**) Similarly, cases with no macroscopic disease were classified by the HRD status for survival rate comparison.

Finally, in the analysis, we included data on residual tumor at the time of surgery. The 514 cases were sorted by tumor size if data on residual tumor at the time of surgery were available, and a poor long-term prognosis for those groups with residual tumors ≥1 mm was revealed regardless of size (Fig. 5A, p < 0.0001). Among the cases with residual tumor ≥1 mm, approximately 25% of genetic HRD and undetermined HRD patients had a long-term survival >7 years, whereas non-HRD and epigenetic HRD patients had low long-term survival rates (Fig. 5B, p = 0.0001). Intriguingly, among patients with no macroscopic diseases, genetic HRD patients had a very high survival rate, and 11 of 12 patients survived after a median observation period of 6.6 years (Fig. 5C, p = 0.0059).

## Discussion

HGSOC data from the TCGA became publicly available in 2011 and have been analyzed in numerous reports since then. However, in this study, we still found some important observations from these data. Our contributions are as follows: (1) establishing a cutoff value to identify HRD from the analysis of HGSOC data alone (2); a comprehensive analysis of HRR pathway genes (3); proposal of a classification method for HRD cases according to cause; and (4) integration of residual tumor data.

A cutoff HRD score ≥42 was previously proposed in a study that combined ovarian and breast cancer SNP array data from the TCGA and two other datasets from custom panels^10^. After running a similar analysis that combined the breast cancer and HGSOC TCGA datasets, *BRCA1/2* mutations seemed to be enriched at HRD scores ≥42 (Supplementary Fig. 1C). However, a comparison of the HGSOC and breast cancer data revealed a large gap in HRD scores (Supplementary Fig. 1A). In another study, a pan-cancer analysis using the TCGA data revealed that the TAI, LST, and LOH scores of HGSOC, with a median of 24, 20, and 15, respectively, were higher than those of cancers derived from other organs; the corresponding median scores for breast cancer were 12, 8, and 8, respectively, for TAI, LST, and LOH, which were approximately half those of HGSOC^13^. Another study calculated the HRD scores of formalin-fixed paraffin-embedded samples using an OncoScan SNP array and found that HGSOC had far higher HRD scores than ovarian clear cell carcinoma, as over 80% of cases scored ≥42^14^. HRD scores thus vary greatly between organ of origin and tissue type. As the aim of our study was to classify HGSOC by HRD status, we based our analyses on HGSOC data only and adopted an HRD score ≥63 as the cutoff. As a result, a little less than half of HGSOC patients were classified in the HRD group (Fig. 1C). In other studies, authors performed SNP arrays with a custom panel, classified a little less than half of their samples as HRD with a cutoff HRD score ≥42, and found a high frequency of *BRCA* mutations^15,16^. The ratio of HRD is consistent with findings from our study even though the cutoff point differs. In order to clarify why such a difference occurred, it would be beneficial if the raw SNP array data that were used to generate the HRD scores for research purposes would be open to the public.

The question of whether HRR pathway gene mutations other than *BRCA1/2* actually cause HRD has not been thoroughly investigated. Our TCGA data analyses indicated that these overall mutations are unrelated to the HRD score (Fig. 2A) and prognosis and that they are not involved in HRD or platinum sensitivity. Among these overall genes, only those cases with *CHEK1* homozygous deletions displayed high HRD scores (Fig. 2B). CHEK1 is required for commencing HRR in cases of DNA double-strand breaks^17^, which means that *CHEK1* homozygous deletions are likely to cause HRD. Moreover, *PTEN* homozygous deletions correlated with high HRD scores (Fig. 2C). Therefore, we suggest that the only genetic changes that are involved in HRD in HGSOC besides the germline/somatic mutations in *BRCA1/2* are *CHEK1* and *PTEN* homozygous deletions. In support of our results, the HRD-associated LOH score is high in ovarian cancer with *PTEN* homozygous deletions, but ovarian cancer cases with *ATM*, *ATR*, *FANCA*, *FANCD2*, *FANCM*, and *PALB2* mutations do not have high LOH scores^7^.

It is well known that HRD is caused by promoter methylation of *BRCA1*^5^; indeed, HRD scores were high in cases in which *BRCA1* was methylated (Fig. 3A). The TCGA database only lists 168 genes including *BRCA1* because of the strict standards for β value and mRNA expression for the selection of methylated genes^4^. In addition, tumors with methylated *RAD51C* also transform to HRD^5^ and have high LOH scores^7^. We focused on the negative correlation between the *RAD51C* β value and gene expression and found, as in previous reports, that *RAD51C* is often methylated in HRD cases (Fig. 3B).

We classified the HRD cases and found that epigenetic HRD cases had a poor prognosis, just like non-HRD cases (Fig. 4B). Previous studies have reported the correlation between *BRCA1* and *RAD51C* methylation and a good prognosis in ovarian cancer^18,19^. However, many recent studies do not support these findings^20–23^. In some cases, methylated *BRCA1* in tumors was found to be demethylated after chemotherapy^24,25^. The presence of *BRCA1* methylation before treatment was associated with post relapse platinum resistance in a study of triple-negative breast cancer^26^. These results suggest that demethylation of methylated *BRCA1* occurs more readily than genetic changes such as the reversion mutation in *BRCA1/2* mutation cases^24,27^. Our findings for the elevated HRD scores in the tumors with *BRCA1* or *RAD51C* methylations (Fig. 3) indicate that there is a poor prognostic subtype in the high-HRD score cases and warrants HRD-positive cases should be classified into epigenetic and non-epigenetic cases when conducting clinical trials.

Compared with genetic HRD cases, non-HRD cases had a poorer prognosis despite the presence of HRD-inducing genetic changes (Supplementary Fig. 3B). Presumably, tumors that managed to regain DNA repair functions through some mechanism became dominant in such cases, even though the DNA repair defects is initially important to carcinogenesis. In HGSOC with *BRCA* abnormalities, tumor infiltration by lymphocytes was augmented^28,29^, which was likely due to tumor immunity triggered by neo-antigen production as a result of DNA repair disorders. In the course of tumor progression, some tumors that regain DNA repair function may become dominant as the tumors interact with immune cells.

Finally, we performed analyses that integrated residual tumors after surgery. The HGSOC TCGA dataset comprises patients who underwent primary debulking surgery. We found that over 90% of genetic HRD patients who had no residual tumor are long-term survivors (Fig. 5C). Consistent with our data, stage III HGSOC cases who survived more than ten years were associated with germline or somatic *BRCA1/2* mutations and no residual disease after primary debulking surgeries^30^. This finding indicates that genetic HRD patients may be those who should undergo the most extensive surgery in order to extend their survival. Presumably, it is possible that tumor cells which harbor genetic alterations like reversion mutations^24,27^ could be minimized in genetic HRD cases by complete resection. Whether or not a similarly high survival rate could be obtained in genetic HRD patients with complete surgery by interval debulking surgery following neoadjuvant chemotherapy remains to be elucidated. Several recent studies have evaluated interval debulking surgery^31,32^; genomic analyses of such clinical study samples would provide the answer to this question.

In conclusion, we classified the HGSOC cases into genetic HRD, epigenetic HRD, undetermined HRD, and non-HRD, each of which had a different prognosis. We found that in the genetic HRD fraction, a high survival rate could be increased by complete resection. Our data could be the foundational basis for future clinical studies in HGSOC.

## Materials and Methods

### Datasets

Raw data from the Affymetrix Genome-Wide Human SNP Array 6.0 and the HT Human Genome U133 Array (Affymetrix) as well as clinical data on HGSOC^4^ and breast invasive carcinoma^12^ were obtained from the GDC Data Portal (https://portal.gdc.cancer.gov). In addition, processed data from Infinium HumanMethylation27 BeadChip (Illumina) was obtained from the GDAC Firehose (http://gdac.broadinstitute.org).

### Data processing

SNP array data of cases for which tumor samples could be matched with a normal sample were extracted. For both tumor and normal sample data, the log R ratio and B-allele frequency of each probe was calculated using Affymetrix Power Tools and PennCNV^33^, and segmented copy number data were generated using allele-specific copy number analysis of tumors^34^. Gene expression data were normalized by the Robust Multi-array Average method using the “affy” package of R.

### HRD score analysis

Genomic scar signatures were analyzed using an R program, as previously described^13^, and TAI, LST, and LOH scores were calculated. Briefly, TAI was defined as the number of regions of allelic imbalance that extended to the telomeres after deciding upon the major copy number state to be used to adjust biases in samples with an uneven chromosome count. LOH was defined as the number of chromosomal LOH regions longer than 15 Mb. LST was defined as the number of break points between regions longer than 10 Mb after filtering out regions shorter than 3 Mb. The HRD score was calculated as the sum of the TAI, LST, and LOH scores. The calculated scores are shown in Supplementary Table 1 (ovarian cancer) and in Supplementary Table 2 (breast cancer).

### Homologous recombination repair (HRR)-related gene analysis

According to the ARIEL3 study and other reports^35,36^, *BRCA1*, *BRCA2*, *ATM*, *ATR*, *BARD1*, *BLM*, *BRIP1*, *CDK12*, *CHEK1*, *CHEK2*, *FANCA*, *FANCC*, *FANCD2*, *FANCE*, *FANCF*, *FANCI*, *FANCL*, *FANCM*, *MRE11*, *NBN*, *PALB2*, *RAD50*, *RAD51*, *RAD51B*, *RAD51C*, *RAD51D*, *RAD52*, *RAD54L*, and *RPA1* were identified as HRR pathway genes. Cases of germline mutations or somatic mutations including homozygous deletions were identified. Moreover, cases of *EMSY* amplification and *PTEN* homozygous deletions, which may cause HRD, and the *CCNE1* amplifications^5^, which are associated with HR proficiency, were identified. These cases were identified using the cBioPortal for Cancer Genomics (http://www.cbioportal.org)^37,38^.

The correlation analysis of HRR pathway genes was performed using promotor methylation DNA microarray data and mRNA expression microarray data. When multiple mRNA expression probes existed, average of these probes was calculated for each case and then normalized from 0 to 1. Subsequently, we selected methylation probes which β values showed strong negative correlation (r < −0.30) with the mRNA expressions. If multiple methylation probes were selected for one gene, the most negatively correlated probe was used.

### Heatmap

A heatmap of the HRD score was created using R 3.2.2 and the MATLAB RGB color specifier. Other heatmaps were drawn using Java TreeView version 1.1.6r4.

### Statistical analysis

We determined the presence of genetic abnormalities, and using the Mann–Whitney U test, we compared the TAI, LST, LOH, and HRD scores between tumors with genetic abnormalities and those without them. We also compared the HRD scores between HGSOC and breast cancer using the Mann-Whitney U test. Correlation analyses were performed using the Pearson correlation coefficient. Survival function was estimated using a Kaplan-Meier analysis, and survival curves were compared between groups using the log-rank test. All statistical analyses were performed using GraphPad Prism 6 (GraphPad Software, San Diego) with an α of 0.05.

## Supplementary information

Supplementary Figure 1, 2, 3, 4, Figure Legends, Table 1, 2.
