# Supplementary material for: Homologous recombination deficiency status-based classification of high-grade serous ovarian carcinoma
Source: Sci Rep. 2020 Feb 17;10:2757. doi: 10.1038/s41598-020-59671-3 (PMC7026096; doi:10.1038/s41598-020-59671-3)
Supplement: Supplementary file 1 — Supplementary Figure 1, 2, 3, 4, Figure Legends, Table 1, 2. [file 41598_2020_59671_MOESM1_ESM.pdf]

Supplementary Data

Title

Homologous recombination deficiency status-based classification of high-grade serous ovarian carcinoma

Authors

Hisamitsu Takaya, Hidekatsu Nakai, Shiro Takamatsu, Masaki Mandai, Noriomi Matsumura

Supplementary Figure 1

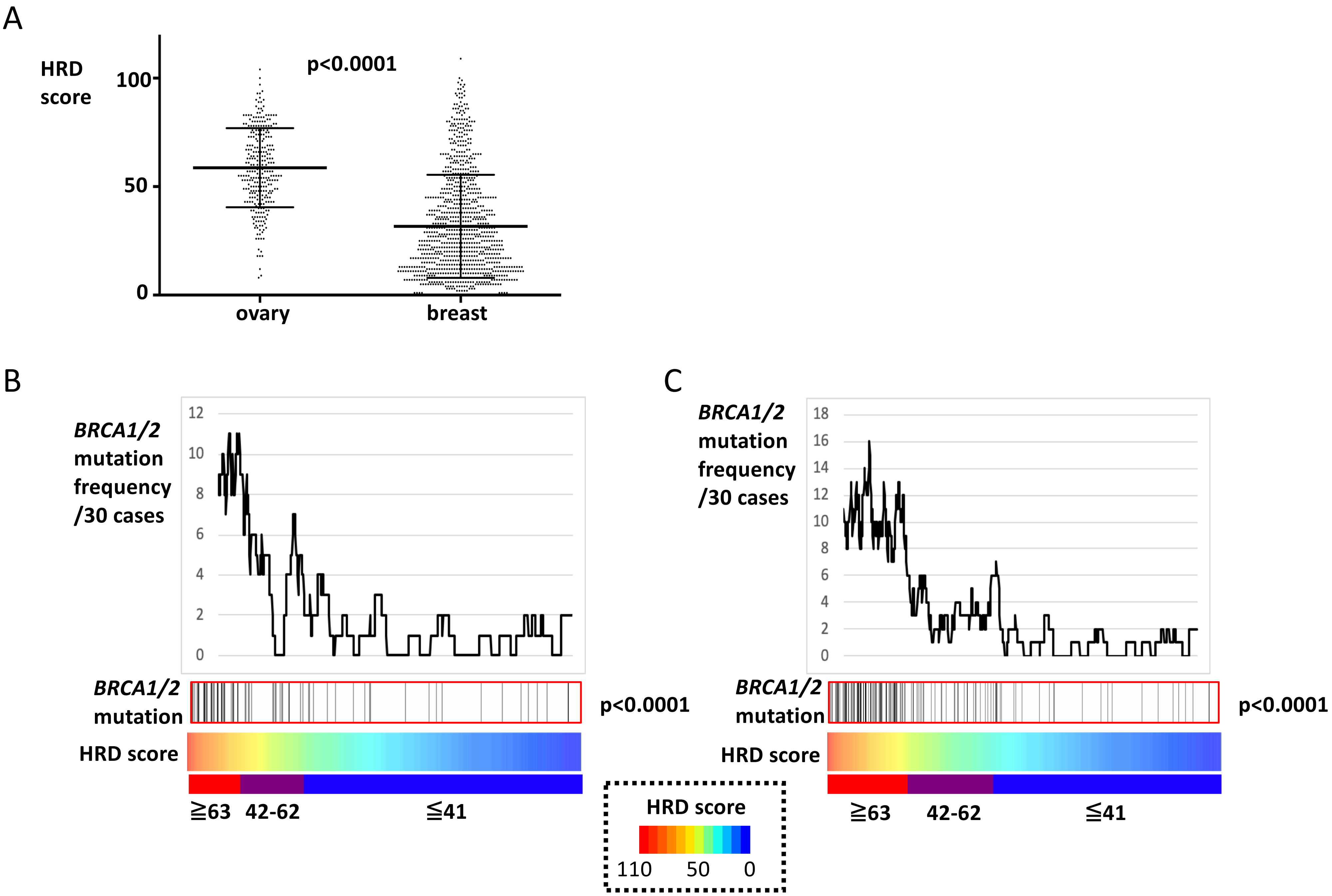

Supplementary Figure 2

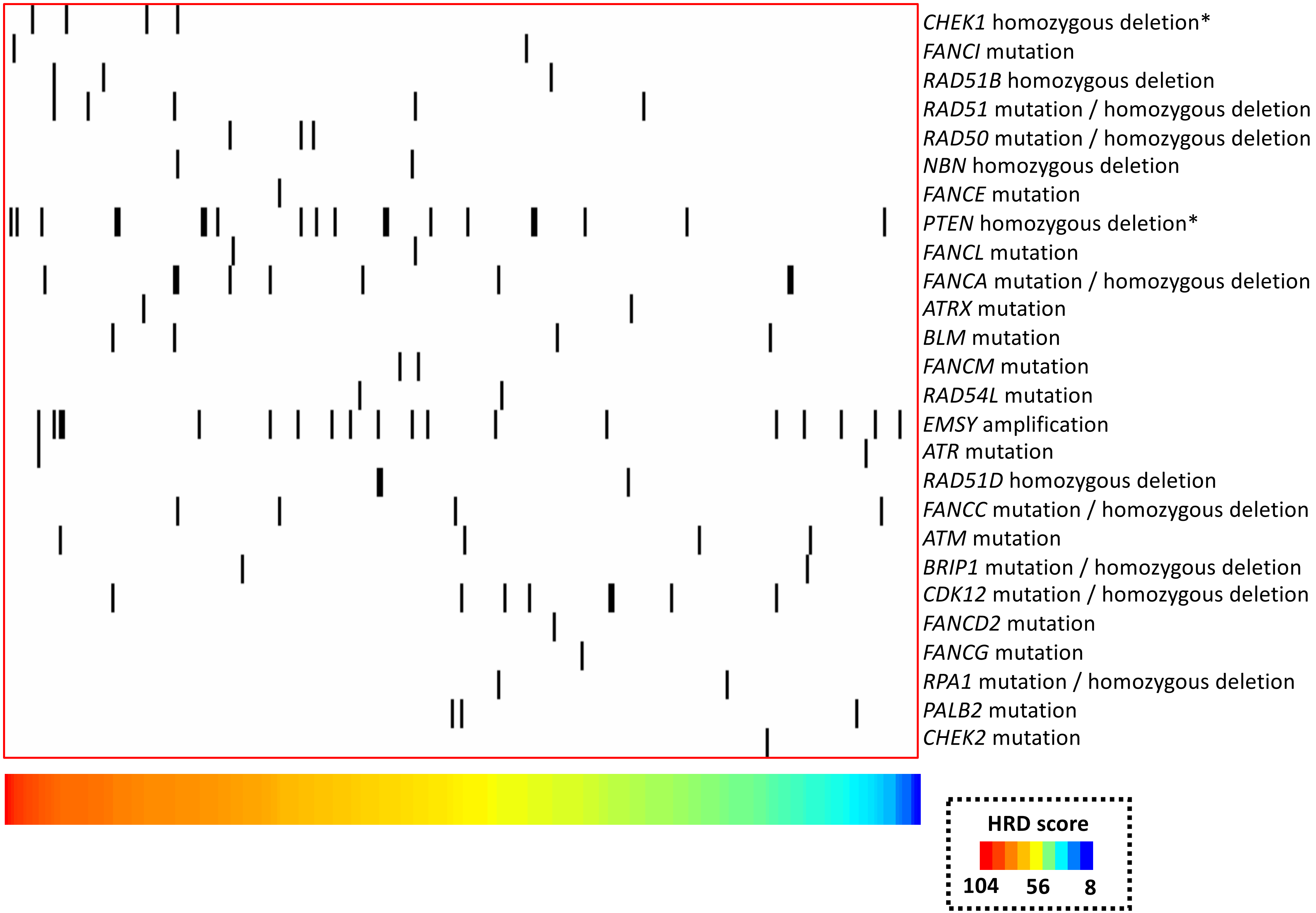

Supplementary Figure 3

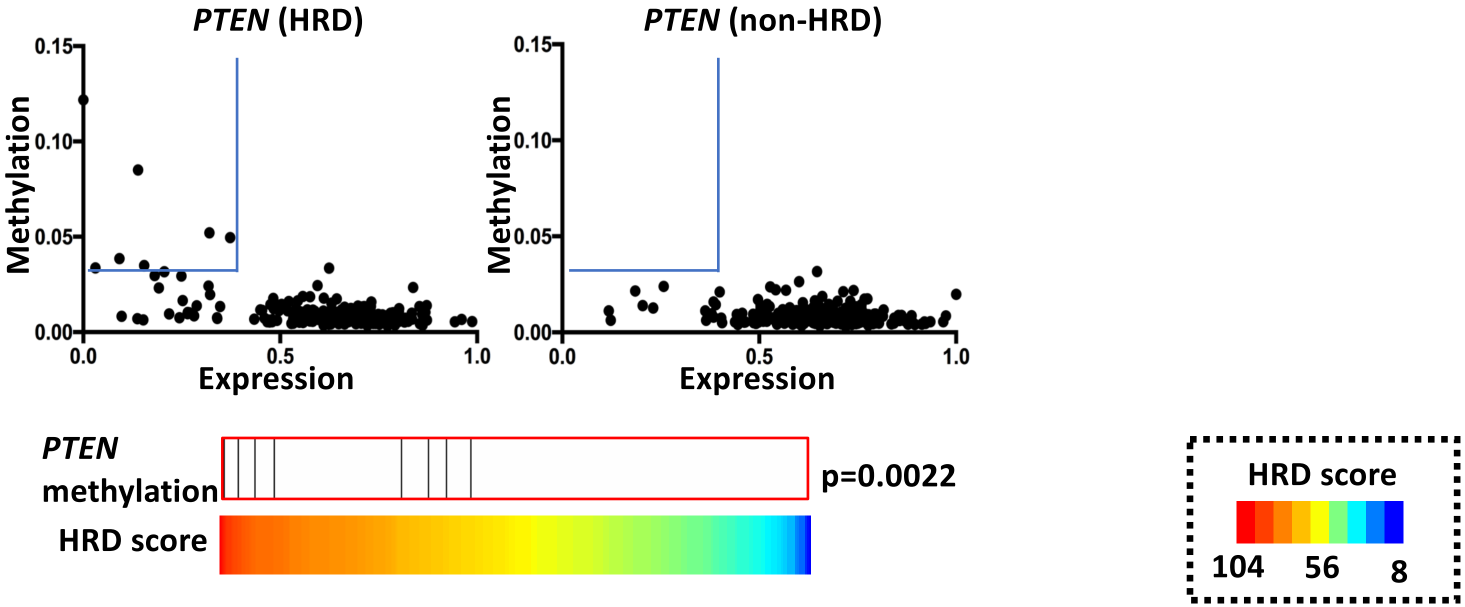

Supplementary Figure 4

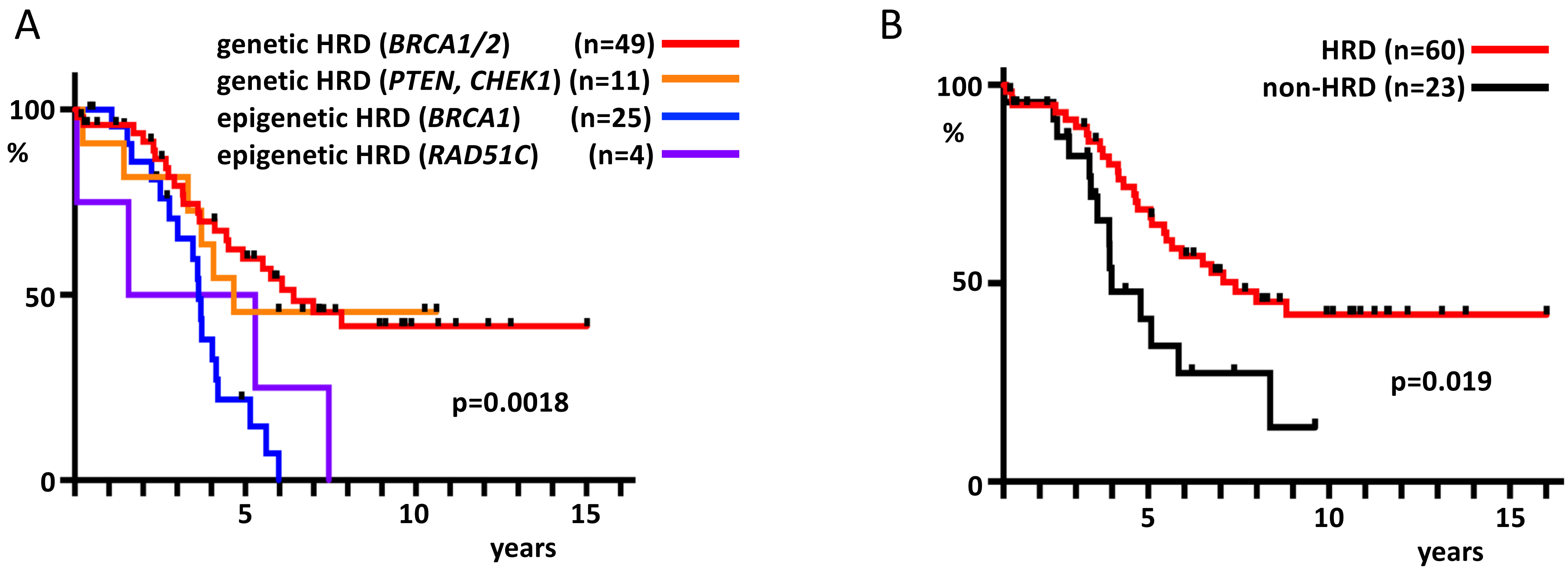

Supplementary Figure Legends

Supplementary Figure 1. Analysis of the breast cancer dataset. A) Comparison of HRD scores. TCGA dataset samples with both exome sequencing and SNP array data (ovary: n = 296; breast: n = 955). B) Distribution of breast cancer *BRCA1/2* mutation cases. The upper graph shows the frequency of *BRCA* mutation cases; TCGA data were sorted by HRD scores using window sliding for 30 cases. The black bars in the central box represent the *BRCA* mutaion cases; the higher the HRD score, the more enriched the *BRCA* mutations. Sections of HRD scores ≥ 63, 42 – 62, and 41 ≥ are shown by colored bars. B) In all, 1251 cases (breast cancer: 955 cases, ovarian cancer: 296 cases) were sorted by HRD score to show the distribution of *BRCA* mutation cases. *BRCA1/2* mutation cases have a bimodal distribution, with an apparent cutoff ≥ 42 as well as ≥ 63.

Supplementary Figure 2. Relationship of HRD scores and HRR pathway gene alterations other than *BRCA1/2*. Gene alterations are sorted by average HRD scores. Only *CHEK1* homozygous deletions and *PTEN* homozygous deletions were significantly enriched in the cases with high HRD scores (*; p=0.0038 and 0.035, respectively).

Supplementary Figure 3. Analysis of *PTEN* methylation. X-axis: average values of three *PTEN* mRNA expression probes normalized to 0 – 1. Y-axis: β value of cg21573601. *PTEN* methylation cases are surrounded by a blue line (upper-left region). The frequency of *PTEN* methylation differs substantially between HRD cases (left) and non-HRD cases (right); β values are generally low.

Supplementary Figure 4. A) Comparison of HRD cases in OS. Genetic HRD cases were divided into those with *BRCA1/2* mutations and those with *CHEK1* or *PTEN* homozygous deletions; epigenetic HRD cases were divided into *BRCA1* methylation cases and *RAD51C* methylation cases. B) Cases with genetic factors (*BRCA*1/2 methylations, *CHEK1* homozygous deletions, and *PTEN* homozygous deletions) were divided into HRD and non-HRD groups for comparison of OS.

Supplementary Table 1. Annotation of TCGA ovarian cancer data (high-grade serous ovarian cancer).

PatientID	HRD score (TAI+LST+LOH)	TAI score	LST score	LOH score	HRD status (≥63; 1, <63; 0)	Classification	OS (days)	Vital status	Residual tumors	*BRCA1* Methylation	*RAD51C* B>0.1, exp<0.2	*PTEN* B>0.03, exp<0.4	*BRCA1*	*BRCA2*	*CCNE1*	*ATM*	*ATR*	*BARD1*	*BLM*	*BRIP1*	*CDK12*	*CHEK1*	*CHEK2*	*FANCA*	*FANCC*	*FANCD2*	*FANCE*	*FANCF*	*FANCI*	*FANCL*	*FANCM*	*MRE11*	*NBN*	*PALB2*	*RAD50*	*RAD51*	*RAD51B*	*RAD51C*	*RAD51D*	*RAD52*	*RAD54L*	*RPA1*	*PTEN*	*EMSY*
TCGA-24-1428	104	36	34	34	1	Epigenetic	529	Alive	1-10 mm	1	0	0	0	0	0	0	0	0	[illegible]	[illegible]	[illegible]	[illegible]	[illegible]	[illegible]	[illegible]	[illegible]	[illegible]	[illegible]	[illegible]	[illegible]	[illegible]	[illegible]	[illegible]	[illegible]	[illegible]	[illegible]	[illegible]	[illegible]	[illegible]	[illegible]	[illegible]	[illegible]	[illegible]	[illegible]
TCGA-10-0928	100	37	38	25	1	Epigenetic	563	Dead	1-10 mm	1	0	0	0	0	0	0	0	0	[illegible]	[illegible]	[illegible]	[illegible]	[illegible]	[illegible]	[illegible]	[illegible]	[illegible]	[illegible]	[illegible]	[illegible]	[illegible]	[illegible]	[illegible]	[illegible]	[illegible]	[illegible]	[illegible]	[illegible]	[illegible]	[illegible]	[illegible]	[illegible]	[illegible]	[illegible]
TCGA-13-1497	97	32	42	23	1	Genetic	3871	Alive		1	0	0	0	0	0	0	0	0	[illegible]	[illegible]	[illegible]	[illegible]	[illegible]	[illegible]	[illegible]	[illegible]	[illegible]	[illegible]	[illegible]	[illegible]	[illegible]	[illegible]	[illegible]	[illegible]	[illegible]	[illegible]	[illegible]	[illegible]	[illegible]	[illegible]	[illegible]	[illegible]	[illegible]	[illegible]
TCGA-13-0923	94	31	47	16	1	Undetermined	2547	Alive	No Macroscopic disease	0	0	0	0	0	0	0	0	0	[illegible]	[illegible]	[illegible]	[illegible]	[illegible]	[illegible]	[illegible]	[illegible]	[illegible]	[illegible]	[illegible]	[illegible]	[illegible]	[illegible]	[illegible]	[illegible]	[illegible]	[illegible]	[illegible]	[illegible]	[illegible]	[illegible]	[illegible]	[illegible]	[illegible]	[illegible]
TCGA-04-1338	93	30	44	19	1	Undetermined	1418	Alive	1-10 mm	0	0	0	0	0	0	0	0	0	[illegible]	[illegible]	[illegible]	[illegible]	[illegible]	[illegible]	[illegible]	[illegible]	[illegible]	[illegible]	[illegible]	[illegible]	[illegible]	[illegible]	[illegible]	[illegible]	[illegible]	[illegible]	[illegible]	[illegible]	[illegible]	[illegible]	[illegible]	[illegible]	[illegible]	[illegible]
TCGA-13-0905	93	27	45	21	1	Genetic	3746	Alive		1	0	1	0	0	0	0	0	0	[illegible]	[illegible]	[illegible]	[illegible]	[illegible]	[illegible]	[illegible]	[illegible]	[illegible]	[illegible]	[illegible]	[illegible]	[illegible]	[illegible]	[illegible]	[illegible]	[illegible]	[illegible]	[illegible]	[illegible]	[illegible]	[illegible]	[illegible]	[illegible]	[illegible]	[illegible]
TCGA-10-0930	91	31	41	19	1	Undetermined	1040	Dead	1-10 mm	0	0	0	0	0	0	0	0	0	[illegible]	[illegible]	[illegible]	[illegible]	[illegible]	[illegible]	[illegible]	[illegible]	[illegible]	[illegible]	[illegible]	[illegible]	[illegible]	[illegible]	[illegible]	[illegible]	[illegible]	[illegible]	[illegible]	[illegible]	[illegible]	[illegible]	[illegible]	[illegible]	[illegible]	[illegible]
TCGA-04-1356	90	31	33	26	1	Genetic	1499	Dead		0	0	0	G-MUT	0	0	0	0	0	[illegible]	[illegible]	[illegible]	[illegible]	[illegible]	[illegible]	[illegible]	[illegible]	[illegible]	[illegible]	[illegible]	[illegible]	[illegible]	[illegible]	[illegible]	[illegible]	[illegible]	[illegible]	[illegible]	[illegible]	[illegible]	[illegible]	[illegible]	[illegible]	[illegible]	[illegible]
TCGA-13-0714	90	30	40	20	1	Undetermined	189	Dead	1-10 mm	0	0	0	0	0	0	0	0	0	[illegible]	[illegible]	[illegible]	[illegible]	[illegible]	[illegible]	[illegible]	[illegible]	[illegible]	[illegible]	[illegible]	[illegible]	[illegible]	[illegible]	[illegible]	[illegible]	[illegible]	[illegible]	[illegible]	[illegible]	[illegible]	[illegible]	[illegible]	[illegible]	[illegible]	[illegible]
TCGA-09-2051	89	30	43	16	1	Genetic	1919	Alive	No Macroscopic disease	0	0	0	G-MUT	0	AMP	0	0	0	[illegible]	[illegible]	[illegible]	[illegible]	[illegible]	[illegible]	[illegible]	[illegible]	[illegible]	[illegible]	[illegible]	[illegible]	[illegible]	[illegible]	[illegible]	[illegible]	[illegible]	[illegible]	[illegible]	[illegible]	[illegible]	[illegible]	[illegible]	[illegible]	[illegible]	[illegible]
TCGA-13-1494	89	27	29	33	1	Genetic	2856	Dead		0	0	0	G-MUT	0	0	0	0	0	[illegible]	[illegible]	[illegible]	[illegible]	[illegible]	[illegible]	[illegible]	[illegible]	[illegible]	[illegible]	[illegible]	[illegible]	[illegible]	[illegible]	[illegible]	[illegible]	[illegible]	[illegible]	[illegible]	[illegible]	[illegible]	[illegible]	[illegible]	[illegible]	[illegible]	[illegible]
TCGA-31-1959	87	28	24	35	1	Genetic	67	Alive		0	0	0	HOMDEL	0	0	0	0	0	[illegible]	[illegible]	[illegible]	[illegible]	[illegible]	[illegible]	[illegible]	[illegible]	[illegible]	[illegible]	[illegible]	[illegible]	[illegible]	[illegible]	[illegible]	[illegible]	[illegible]	[illegible]	[illegible]	[illegible]	[illegible]	[illegible]	[illegible]	[illegible]	[illegible]	[illegible]
TCGA-13-0760	87	30	37	20	1	Undetermined	351	Dead	No Macroscopic disease	0			0	0	0	0	MUT	0	[illegible]	[illegible]	[illegible]	[illegible]	[illegible]	[illegible]	[illegible]	[illegible]	[illegible]	[illegible]	[illegible]	[illegible]	[illegible]	[illegible]	[illegible]	[illegible]	[illegible]	[illegible]	[illegible]	[illegible]	[illegible]	[illegible]	[illegible]	[illegible]	[illegible]	[illegible]
TCGA-23-1117	86	34	31	21	1	Epigenetic	1013	Dead	1-10 mm	1	0	0	0	0	0	0	0	0	[illegible]	[illegible]	[illegible]	[illegible]	[illegible]	[illegible]	[illegible]	[illegible]	[illegible]	[illegible]	[illegible]	[illegible]	[illegible]	[illegible]	[illegible]	[illegible]	[illegible]	[illegible]	[illegible]	[illegible]	[illegible]	[illegible]	[illegible]	[illegible]	[illegible]	[illegible]
TCGA-24-2290	86	28	39	19	1	Epigenetic	1102	Dead	1-10 mm	1	0	0	0	0	0	0	0	0	[illegible]	[illegible]	[illegible]	[illegible]	[illegible]	[illegible]	[illegible]	[illegible]	[illegible]	[illegible]	[illegible]	[illegible]	[illegible]	[illegible]	[illegible]	[illegible]	[illegible]	[illegible]	[illegible]	[illegible]	[illegible]	[illegible]	[illegible]	[illegible]	[illegible]	[illegible]
TCGA-23-2078	85	29	31	25	1	Genetic	2661	Alive	No Macroscopic disease	0	0	0	G-MUT	0	0	0	0	0	[illegible]	[illegible]	[illegible]	[illegible]	[illegible]	[illegible]	[illegible]	[illegible]	[illegible]	[illegible]	[illegible]	[illegible]	[illegible]	[illegible]	[illegible]	[illegible]	[illegible]	[illegible]	[illegible]	[illegible]	[illegible]	[illegible]	[illegible]	[illegible]	[illegible]	[illegible]
TCGA-13-0887	84	26	35	23	1	Undetermined	2028	Dead	1-10 mm	0	0	0	0	0	0	0	0	0	[illegible]	[illegible]	[illegible]	[illegible]	[illegible]	[illegible]	[illegible]	[illegible]	[illegible]	[illegible]	[illegible]	[illegible]	[illegible]	[illegible]	[illegible]	[illegible]	[illegible]	[illegible]	[illegible]	[illegible]	[illegible]	[illegible]	[illegible]	[illegible]	[illegible]	[illegible]
TCGA-13-0904	84	28	34	22	1	Epigenetic	1532	Dead	>20 mm	1	0	0	0	0	0	0	0	0	[illegible]	[illegible]	[illegible]	[illegible]	[illegible]	[illegible]	[illegible]	[illegible]	[illegible]	[illegible]	[illegible]	[illegible]	[illegible]	[illegible]	[illegible]	[illegible]	[illegible]	[illegible]	[illegible]	[illegible]	[illegible]	[illegible]	[illegible]	[illegible]	[illegible]	[illegible]
TCGA-13-1489	83	32	26	25	1	Genetic	2553	Dead		0	0	0	MUT	0	0	AMP		0	[illegible]	[illegible]	[illegible]	[illegible]	[illegible]	[illegible]	[illegible]	[illegible]	[illegible]	[illegible]	[illegible]	[illegible]	[illegible]	[illegible]	[illegible]	[illegible]	[illegible]	[illegible]	[illegible]	[illegible]	[illegible]	[illegible]	[illegible]	[illegible]	[illegible]	[illegible]
TCGA-23-2079	83	30	27	26	1	Genetic	2788	Alive	No Macroscopic disease	0	0	0	G-MUT	0	0	0	0	0	[illegible]	[illegible]	[illegible]	[illegible]	[illegible]	[illegible]	[illegible]	[illegible]	[illegible]	[illegible]	[illegible]	[illegible]	[illegible]	[illegible]	[illegible]	[illegible]	[illegible]	[illegible]	[illegible]	[illegible]	[illegible]	[illegible]	[illegible]	[illegible]	[illegible]	[illegible]
TCGA-25-2401	83	28	37	18	1	Genetic	90	Dead	1-10 mm	0	0	0	G-MUT	HOMDEL	0	0	0	0	[illegible]	[illegible]	[illegible]	[illegible]	[illegible]	[illegible]	[illegible]	[illegible]	[illegible]	[illegible]	[illegible]	[illegible]	[illegible]	[illegible]	[illegible]	[illegible]	[illegible]	[illegible]	[illegible]	[illegible]	[illegible]	[illegible]	[illegible]	[illegible]	[illegible]	[illegible]
TCGA-09-2050	83	26	35	22	1	Genetic	2163	Alive	No Macroscopic disease	0	0	0	0	MUT	0	0	0	0	[illegible]	[illegible]	[illegible]	[illegible]	[illegible]	[illegible]	[illegible]	[illegible]	[illegible]	[illegible]	[illegible]	[illegible]	[illegible]	[illegible]	[illegible]	[illegible]	[illegible]	[illegible]	[illegible]	[illegible]	[illegible]	[illegible]	[illegible]	[illegible]	[illegible]	[illegible]
TCGA-13-0916	83	30	22	31	1	Epigenetic	1785	Alive	No Macroscopic disease	1	0	0	0	AMP	AMP	0	0	0	AMP	[illegible]	[illegible]	[illegible]	[illegible]	[illegible]	[illegible]	[illegible]	[illegible]	[illegible]	[illegible]	[illegible]	[illegible]	[illegible]	[illegible]	[illegible]	[illegible]	[illegible]	[illegible]	[illegible]	[illegible]	[illegible]	[illegible]	[illegible]	[illegible]	[illegible]
TCGA-13-1501	83	29	37	17	1	Epigenetic	1314	Dead	>20 mm	1	0	1	0	0	0	MUT	0	0	[illegible]	[illegible]	[illegible]	[illegible]	[illegible]	[illegible]	[illegible]	[illegible]	[illegible]	[illegible]	[illegible]	[illegible]	[illegible]	[illegible]	[illegible]	[illegible]	[illegible]	[illegible]	[illegible]	[illegible]	[illegible]	[illegible]	[illegible]	[illegible]	[illegible]	[illegible]
TCGA-24-1614	83	28	29	26	1	Epigenetic	1470	Dead	1-10 mm	1	0	0	0	0	AMP	0	0	0	[illegible]	[illegible]	[illegible]	[illegible]	[illegible]	[illegible]	[illegible]	[illegible]	[illegible]	[illegible]	[illegible]	[illegible]	[illegible]	[illegible]	[illegible]	[illegible]	[illegible]	[illegible]	[illegible]	[illegible]	[illegible]	[illegible]	[illegible]	[illegible]	[illegible]	[illegible]
TCGA-24-2254	83	28	33	22	1	Undetermined	1736	Dead	1-10 mm	0	0	0	0	0	0	0	0	0	[illegible]	[illegible]	[illegible]	[illegible]	[illegible]	[illegible]	[illegible]	[illegible]	[illegible]	[illegible]	[illegible]	[illegible]	[illegible]	[illegible]	[illegible]	[illegible]	[illegible]	[illegible]	[illegible]	[illegible]	[illegible]	[illegible]	[illegible]	[illegible]	[illegible]	[illegible]
TCGA-36-1578	83	31	34	18	1	Undetermined	847	Alive	>20 mm	0	0	0	0	0	0	0	0	0	[illegible]	[illegible]	[illegible]	[illegible]	[illegible]	[illegible]	[illegible]	[illegible]	[illegible]	[illegible]	[illegible]	[illegible]	[illegible]	[illegible]	[illegible]	[illegible]	[illegible]	[illegible]	[illegible]	[illegible]	[illegible]	[illegible]	[illegible]	[illegible]	[illegible]	[illegible]
TCGA-13-0890	82	29	22	31	1	Genetic	4424	Alive	1-10 mm	0	0	0	0	MUT	0	0	0	0	[illegible]	[illegible]	[illegible]	[illegible]	[illegible]	[illegible]	[illegible]	[illegible]	[illegible]	[illegible]	[illegible]	[illegible]	[illegible]	[illegible]	[illegible]	[illegible]	[illegible]	[illegible]	[illegible]	[illegible]	[illegible]	[illegible]	[illegible]	[illegible]	[illegible]	[illegible]
TCGA-23-1028	82	33	29	20	1	Undetermined	1503	Alive	1-10 mm	0	0	0	0	0	0	0	0	0	[illegible]	[illegible]	[illegible]	[illegible]	[illegible]	[illegible]	[illegible]	[illegible]	[illegible]	[illegible]	[illegible]	[illegible]	[illegible]	[illegible]	[illegible]	[illegible]	[illegible]	[illegible]	[illegible]	[illegible]	[illegible]	[illegible]	[illegible]	[illegible]	[illegible]	[illegible]
TCGA-24-1427	82	29	27	26	1	Undetermined	147	Alive	>20 mm	0	0	0	0	0	0	0	0	0	[illegible]	[illegible]	[illegible]	[illegible]	[illegible]	[illegible]	[illegible]	[illegible]	[illegible]	[illegible]	[illegible]	[illegible]	[illegible]	[illegible]	[illegible]	[illegible]	[illegible]	[illegible]	[illegible]	[illegible]	[illegible]	[illegible]	[illegible]	[illegible]	[illegible]	[illegible]
TCGA-24-1435	82	28	39	15	1	Epigenetic	1324	Dead	1-10 mm	1		0	0	0	0	0	0	0	[illegible]	[illegible]	[illegible]	[illegible]	[illegible]	[illegible]	[illegible]	[illegible]	[illegible]	[illegible]	[illegible]	[illegible]	[illegible]	[illegible]	[illegible]	[illegible]	[illegible]	[illegible]	[illegible]	[illegible]	[illegible]	[illegible]	[illegible]	[illegible]	[illegible]	[illegible]
TCGA-36-1577	82	29	26	27	1	Undetermined	783	Alive	No Macroscopic disease	0	0	0	0	0	0	0	0	0	[illegible]	[illegible]	[illegible]	[illegible]	[illegible]	[illegible]	[illegible]	[illegible]	[illegible]	[illegible]	[illegible]	[illegible]	[illegible]	[illegible]	[illegible]	[illegible]	[illegible]	[illegible]	[illegible]	[illegible]	[illegible]	[illegible]	[illegible]	[illegible]	[illegible]	[illegible]
TCGA-04-1530	81	27	40	14	1	Undetermined	3622	Dead	1-10 mm	0	0	0	0	0	0	0	AMP	0	[illegible]	[illegible]	[illegible]	[illegible]	[illegible]	[illegible]	[illegible]	[illegible]	[illegible]	[illegible]	[illegible]	[illegible]	[illegible]	[illegible]	[illegible]	[illegible]	[illegible]	[illegible]	[illegible]	[illegible]	[illegible]	[illegible]	[illegible]	[illegible]	[illegible]	[illegible]
TCGA-09-2044	81	31	31	19	1	Epigenetic	186	Alive	No Macroscopic disease	1	0	0	0	0	0	0	0	0	[illegible]	[illegible]	[illegible]	[illegible]	[illegible]	[illegible]	[illegible]	[illegible]	[illegible]	[illegible]	[illegible]	[illegible]	[illegible]	[illegible]	[illegible]	[illegible]	[illegible]	[illegible]	[illegible]	[illegible]	[illegible]	[illegible]	[illegible]	[illegible]	[illegible]	[illegible]
TCGA-24-2288	81	28	29	24	1	Genetic	25	Dead	>20 mm	0	0	0	0	G-MUT	0	0	0	0	[illegible]	[illegible]	[illegible]	[illegible]	[illegible]	[illegible]	[illegible]	[illegible]	[illegible]	[illegible]	[illegible]	[illegible]	[illegible]	[illegible]	[illegible]	[illegible]	[illegible]	[illegible]	[illegible]	[illegible]	[illegible]	[illegible]	[illegible]	[illegible]	[illegible]	[illegible]
TCGA-25-1630	80	22	30	28	1	Genetic	1162	Dead	1-10 mm	0	0	0	MUT	0	0	AMP		0	[illegible]	[illegible]	[illegible]	[illegible]	[illegible]	[illegible]	[illegible]	[illegible]	[illegible]	[illegible]	[illegible]	[illegible]	[illegible]	[illegible]	[illegible]	[illegible]	[illegible]	[illegible]	[illegible]	[illegible]	[illegible]	[illegible]	[illegible]	[illegible]	[illegible]	[illegible]
[illegible]																																												

TCGA-13-0799	79	29	31	19	1	2535	Alive	No Macroscopic disease	0	0	0
TCGA-13-0801	86	29	37	20	1	1399	Dead	No Macroscopic disease	0	0	1
TCGA-13-0802	38	16	15	7	0	1552	Dead	No Macroscopic disease	0	0	0
TCGA-13-0803	59	23	28	8	0	1334	Dead	No Macroscopic disease	0	0	0
TCGA-13-0805	56	20	20	16	0	1074	Dead	1-10 mm	0	0	0
TCGA-13-0888	48	34	14	0	0	2811	Dead	No Macroscopic disease	0	0	0
TCGA-13-0901	40	20	14	6	0	589	Alive	>20 mm	0	0	0
TCGA-13-0908	78	27	36	15	1	1843	Dead	1-10 mm	1	0	0
TCGA-13-0913-02											
TCGA-13-0921	48	25	18	5	0	1483	Dead	No Macroscopic disease	0	0	0
TCGA-13-1485	23	11	9	3	0	629	Dead	1-10 mm	0	0	0
TCGA-13-1489-02											
TCGA-13-1500	50	22	18	10	0	425	Dead	11-20 mm	0	0	0
TCGA-13-1511	38	16	15	7	0	1650	Dead	>20 mm	0	0	0
TCGA-13-1817						1024	Dead	>20 mm		0	0
TCGA-13-1817-02											
TCGA-13-1819						2025	Dead	No Macroscopic disease		0	0
TCGA-13-1819-02											
TCGA-13-2057						2434	Alive	No Macroscopic disease			
TCGA-13-2059	91	34	30	27	1	2387	Alive	1-10 mm			
TCGA-13-2061	78	33	28	17	1	2339	Alive	No Macroscopic disease			
TCGA-13-2065	77	25	26	26	1	1993	Alive	1-10 mm			
TCGA-13-2066						2133	Dead	1-10 mm			
TCGA-13-2071	93	32	39	22	1	773	Dead	1-10 mm			
TCGA-13-A5FT	46	21	16	9	0	2143	Alive	No Macroscopic disease			
TCGA-13-A5FU	50	19	24	7	0	256	Dead	1-10 mm			
TCGA-20-0996						2196	Alive	1-10 mm		0	0
TCGA-20-1682	78	30	32	16	1	837	Alive	11-20 mm	0	0	0
TCGA-20-1683	42	24	11	7	0	772	Alive	No Macroscopic disease	0	0	0
TCGA-20-1684	40	24	10	6	0	581	Alive	1-10 mm	0	0	0
TCGA-20-1685	71	26	30	15	1	508	Alive	1-10 mm	0	0	0
TCGA-20-1686	38	17	16	5	0	89	Alive	No Macroscopic disease	0	0	0
TCGA-20-1687	74	27	31	16	1	81	Alive	No Macroscopic disease	0	0	0
TCGA-23-1029	65	26	30	9	1	268	Alive	1-10 mm	0	0	0
TCGA-23-1107						9	Dead	>20 mm	0	0	0
TCGA-23-1109	47	19	23	5	0	1562	Dead	1-10 mm	0	0	0
TCGA-23-1111	40	25	13	2	0	98	Alive	>20 mm	0	0	0
TCGA-23-1113	26	11	12	3	0	949	Dead	1-10 mm	0	0	0
TCGA-23-1114	58	26	28	4	0	2089	Dead	No Macroscopic disease	0	0	0
TCGA-23-1119						3953	Alive	1-10 mm	0	0	0
TCGA-23-1121	77	34	28	15	1	194	Alive	>20 mm	0	0	0
TCGA-23-1809	70	31	23	16	1	16	Alive	1-10 mm	0	0	0
TCGA-23-2084	65	28	28	9	1	1516	Dead	No Macroscopic disease	0	0	0
TCGA-23-2641	77	24	32	21	1	84	Alive	1-10 mm			
TCGA-23-2643	42	17	15	10	0	143	Alive	1-10 mm			
TCGA-23-2645	75	27	31	17	1	137	Alive	No Macroscopic disease			
TCGA-23-2647						135	Alive	1-10 mm			
TCGA-23-2649	72	26	32	14	1	116	Alive	1-10 mm			
TCGA-24-1430	80	27	27	26	1	863	Dead	>20 mm	1	0	0
TCGA-24-1467	58	24	24	10	0	3224	Dead	1-10 mm	0	0	0
TCGA-24-1546	81	26	29	26	1	1955	Dead	1-10 mm	0	0	0
TCGA-24-1550	81	30	26	25	1	1249	Dead	1-10 mm	0	0	0
TCGA-24-1842	54	24	20	10	0	253	Alive	1-10 mm	0	0	0
TCGA-24-1843	38	19	16	3	0	106	Alive	1-10 mm	0	0	0
TCGA-24-1844	62	27	31	4	0	113	Alive	1-10 mm	0	0	0
TCGA-24-1845	97	34	43	20	1	116	Alive	1-10 mm	1	0	0
TCGA-24-1846	65	21	21	23	1	133	Alive	1-10 mm	0	0	0
TCGA-24-1847	80	25	41	14	1	343	Alive	1-10 mm	0	0	0
TCGA-24-1849	59	23	36	0	0	176	Alive	1-10 mm	0	0	0
TCGA-24-1850	62	22	22	18	0	168	Alive	1-10 mm	0	0	0
TCGA-24-1852	46	22	16	8	0	2201	Dead	1-10 mm		0	0
TCGA-24-1852-02											
TCGA-24-1920	45	30	7	8	0	298	Alive	1-10 mm	0	0	0
TCGA-24-1923	56	20	16	20	0	690	Dead	1-10 mm	0	0	0
TCGA-24-1924	66	22	34	10	1	919	Dead	1-10 mm	0	1	0
TCGA-24-1927	71	28	29	14	1	500	Dead	1-10 mm		0	0
TCGA-24-1928	36	18	12	6	0	336	Dead	11-20 mm	0	0	0
TCGA-24-1930	66	24	31	11	1	2467	Dead	1-10 mm	0	0	0
TCGA-24-2020	80	33	22	25	1	4624	Dead	1-10 mm	0	0	0
TCGA-24-2023	37	20	8	9	0	1364	Dead	1-10 mm	0	0	0
TCGA-24-2026						1059	Dead	>20 mm	0	0	0
TCGA-24-2027	62	24	22	16	0	3337	Dead	>20 mm	0	0	0
TCGA-24-2029	49	20	20	9	0	439	Dead	>20 mm	0	0	0
TCGA-24-2033	59	25	28	6	0	562	Dead	>20 mm	0	0	0
TCGA-24-2036	65	28	24	13	1	1947	Dead	1-10 mm	0	0	1
TCGA-24-2295	50	25	22	3	0	1007	Dead	>20 mm		0	0
TCGA-24-2297	69	27	22	20	1	1699	Dead	>20 mm	0	0	1
TCGA-25-1312	50	22	21	7	0	31	Dead	1-10 mm	0	0	0
TCGA-25-1314	63	25	26	12	1	1004	Dead	1-10 mm	0	0	0
TCGA-25-1323						395	Dead	1-10 mm	0	0	0
TCGA-25-1325						976	Dead	>20 mm	0	1	0
TCGA-25-1870						455	Dead	11-20 mm	0	0	0
TCGA-25-1871	56	29	22	5	0	760	Dead	11-20 mm	0	0	0
TCGA-25-1877	49	22	19	8	0	730	Dead	1-10 mm	0	0	0
TCGA-25-1878	71	33	27	11	1	2587	Dead	1-10 mm	0	0	0
TCGA-25-2390											
TCGA-25-2397	88	33	33	22	1	365	Dead	No Macroscopic disease	1	0	0
TCGA-29-1688	83	31	35	17	1	2400	Dead		0	0	0
TCGA-29-1690	27	14	12	1	0	1448	Dead	11-20 mm	0	0	0
TCGA-29-1691	96	34	38	24	1	1470	Dead	1-10 mm	0	0	0
TCGA-29-1692						2872	Dead	11-20 mm		0	0
TCGA-29-1692-02											
TCGA-29-1693	71	32	23	16	1	3096	Alive	>20 mm	0	0	0
TCGA-29-1694	51	21	17	13	0	1187	Dead	11-20 mm	1	0	0
TCGA-29-1695	47	20	25	2	0	1229	Dead	>20 mm	0	0	0
TCGA-29-1696	58	20	25	13	0	1032	Dead	1-10 mm	0	0	0
TCGA-29-1697	40	18	12	10	0	949	Dead	>20 mm	0	0	0
TCGA-29-1698						2078	Alive	1-10 mm	0	0	0
TCGA-29-1699						1106	Dead	1-10 mm	0	0	0
TCGA-29-1701	50	18	21	11	0	515	Dead		0	0	0
TCGA-29-1702	53	22	22	9	0	728	Dead	1-10 mm	0	0	0
TCGA-29-1703	39	17	16	6	0	1815	Dead	1-10 mm	0	0	0
TCGA-29-1704	62	19	27	16	0	1587	Alive	11-20 mm		0	0
TCGA-29-1705	75	24	30	21	1	555	Dead	>20 mm	1	0	0
TCGA-29-1705-02											
TCGA-29-1707	64	27	22	15	1	1277	Alive	No Macroscopic disease	1	0	0
TCGA-29-1707-02											
TCGA-29-1710	42	21	15	6	0	951	Dead	1-10 mm	0	0	0
TCGA-29-1710-02											
TCGA-29-1711	81	26	36	19	1	1053	Alive	No Macroscopic disease	1	0	0
TCGA-29-1761	46	21	16	9	0	528	Dead	>20 mm	0	0	0
TCGA-29-1762	46	20	13	13	0	2634	Dead	>20 mm	0	0	0
TCGA-29-1763	93	32	39	22	1	2032	Alive	1-10 mm	0	0	0
TCGA-29-1764	65	24	28	13	1	1914	Alive	>20 mm	0	0	0
TCGA-29-1766	58	28	23	7	0	1199	Dead	11-20 mm	0	0	0
TCGA-29-1768	65	20	24	21	1	952	Dead	>20 mm	0	0	0
TCGA-29-1769	66	25	24	17	1	699	Alive	1-10 mm	1	0	0
TCGA-29-1770	77	28	31	18	1	741	Alive	1-10 mm	0	0	0
TCGA-29-1770-02											
TCGA-29-1771	26	12	10	4	0	686	Alive	>20 mm	0	0	0
TCGA-29-1774	49	20	18	11	0	527	Alive	>20 mm	0	0	0
TCGA-29-1775	64	28	23	13	1	376	Alive	1-10 mm	0	0	0
TCGA-29-1776	56	19	22	15	0	360	Alive	>20 mm	0	0	0
TCGA-29-1777	79	32	26	21	1	374	Alive	>20 mm	1	0	0
TCGA-29-1778	53	26	21	6	0	454	Alive	No Macroscopic disease	0	0	0
TCGA-29-1781	83	30	32	21	1	255	Alive	No Macroscopic disease	0	0	0
TCGA-29-1783	34	14	15	5	0	220	Alive	1-10 mm	0	0	0
TCGA-29-1784	78	29	37	12	1	163	Alive	No Macroscopic disease	0	0	0
TCGA-29-1785	56	24	24	8	0	1104	Dead	>20 mm	0	0	0
TCGA-29-2414						2621	Dead	11-20 mm	0	0	0
TCGA-29-2414-02											
TCGA-29-2425	73	25	37	11	1	1977	Alive	>20 mm	0	0	0
TCGA-29-2428	45	23	17	5	0	1372	Alive	>20 mm	0	0	0
TCGA-29-2429	71	29	26	16	1	421	Dead	>20 mm		0	0
TCGA-29-2431	45	19	20	6	0	1285	Alive	1-10 mm			
TCGA-29-2432	70	30	29	11	1	653	Dead	11-20 mm			
TCGA-29-2434	92	30	34	28	1	349	Alive	No Macroscopic disease			
TCGA-29-2435											
TCGA-29-2436	36	15	14	7	0	297	Alive	1-10 mm			
TCGA-29-A5NZ	45	22	20	3	0	1088	Dead				
TCGA-30-1714	47	25	22	0	0	1158	Dead	1-10 mm	0	0	0
TCGA-30-1718	77	29	37	11	1	1579	Dead	1-10 mm	0	0	0
TCGA-30-1855	67	25	24	18	1	75	Dead	1-10 mm	0	1	0
TCGA-30-1856	47	18	24	5	0	477	Dead	1-10 mm	0	0	0
TCGA-30-1857	76	26	33	17	1	8	Dead		0	0	0
TCGA-30-1859	37	19	15	3	0	1463	Dead	1-10 mm	0	0	0
TCGA-30-1860	110	30	43	37	1	1366	Dead	>20 mm	1	0	1
TCGA-30-1861	77	28	38	11	1	1058	Dead	1-10 mm	1	0	0
TCGA-30-1866	37	17	17	3	0	1114	Dead	1-10 mm	0	0	0
TCGA-30-1867	68	33	26	9	1	37	Dead		0	0	0
TCGA-30-1869											
TCGA-30-1880	42	19	19	4	0	1751	Dead	No Macroscopic disease	0	0	0
TCGA-30-1887	55	26	16	13	0	738	Dead	1-10 mm	0	0	0
TCGA-30-1892	45	20	15	10	0	1484	Dead	1-10 mm	0	0	0
TCGA-31-1944	61	22	27	12	0	1386	Alive	No Macroscopic disease	0	0	0
TCGA-31-1946	62	25	23	14	0	918	Alive	No Macroscopic disease	1	0	0
TCGA-31-1951	47	20	18	9	0	684	Alive	No Macroscopic disease	0	0	0
TCGA-31-1955	58	19	22	17	0	1293	Alive	No Macroscopic disease		0	0
TCGA-31-1956	56	21	19	16	0	1342	Alive		0	0	0
TCGA-36-1581	59	27	23	9	0	751	Alive	No Macroscopic disease	0	0	0
TCGA-36-2529	48	20	23	5	0	1066	Alive	>20 mm			
TCGA-36-2530	78	29	32	17	1	1004	Alive	11-20 mm			
TCGA-36-2532	56	19	23	14	0	897	Alive	No Macroscopic disease			
TCGA-36-2533	75	27	36	12	1						
TCGA-36-2534	86	28	31	27	1	907	Alive	>20 mm			
TCGA-36-2537	65	24	27	14	1	788	Alive	>20 mm			
TCGA-36-2538	76	27	23	26	1	763	Alive	11-20 mm			
TCGA-36-2539	83	23	31	29	1						
TCGA-36-2540	34	10	13	11	0	724	Alive	>20 mm			
TCGA-36-2542	64	24	24	16	1	657	Alive	1-10 mm			
TCGA-36-2543	84	29	30	25	1	68	Dead	>20 mm			
TCGA-36-2544	84	30	36	18	1	638	Alive	>20 mm			
TCGA-36-2545	68	29	28	11	1	557	Alive	No Macroscopic disease			
TCGA-36-2547	78	34	29	15	1	624	Alive	No Macroscopic disease			
TCGA-36-2548	52	23	25	4	0	431	Alive	>20 mm			
TCGA-36-2549	79	24	39	16	1	184	Alive	No Macroscopic disease			
TCGA-36-2551						492	Alive	>20 mm			
TCGA-36-2552	68	27	26	15	1	349	Alive	No Macroscopic disease			
TCGA-3P-A9WA	61	24	23	14	0	420	Alive				
TCGA-42-2582	57	22	24	11	0	1447	Dead	No Macroscopic disease			
TCGA-42-2587	42	12	30	0	0	722	Alive	>20 mm			
TCGA-42-2588	52	24	21	7	0	880	Dead	>20 mm			
TCGA-42-2589	79	31	29	19	1	1278	Alive	1-10 mm			
TCGA-42-2590	72	24	36	12	1	317	Dead	>20 mm			
TCGA-42-2591	91	30	31	30	1	1240	Alive	No Macroscopic disease			
TCGA-42-2593	59	23	23	13	0	45	Dead	>20 mm			
TCGA-57-1585	75	28	30	17	1	53	Dead	11-20 mm	1	0	0
TCGA-57-1586	47	22	15	10	0	679	Alive	11-20 mm	0	0	0
TCGA-57-1992	57	19	18	20	0	882	Alive	No Macroscopic disease		0	0
TCGA-57-1994	73	31	26	16	1	761	Alive		0	0	0
TCGA-59-2349	24	8	13	3	0	2437	Dead	No Macroscopic disease		0	0
TCGA-59-2372	59	25	25	9	0	1363	Dead	No Macroscopic disease			
TCGA-59-A5PD	91	31	34	26	1	624	Dead				
TCGA-5X-AA5U	55	27	22	6	0	361	Alive				
TCGA-61-1721	18	7	10	1	0	338	Alive		0	0	0
TCGA-61-1722	64	20	21	23	1	246	Alive	No Macroscopic disease		0	0
TCGA-61-1724	85	33	27	25	1	637	Dead	No Macroscopic disease	1	0	0
TCGA-61-1725	71	25	26	20	1	956	Alive	11-20 mm	0	0	0
TCGA-61-1727	68	27	30	11	1	564	Alive	No Macroscopic disease		0	0
TCGA-61-1730	47	26	15	6	0	132	Dead	1-10 mm		0	0
TCGA-61-1733	42	18	17	7	0	967	Alive	No Macroscopic disease	0	0	0
TCGA-61-1734	78	26	25	27	1	866	Alive			0	0
TCGA-61-1737	78	25	32	21	1	1364	Alive	1-10 mm	0	0	0
TCGA-61-1738	44	20	17	7	0	1089	Dead	1-10 mm	0	0	0
TCGA-61-1740	92	29	36	27	1	74	Dead	>20 mm	0	0	0
TCGA-61-1741	48	25	16	7	0	1024	Dead	>20 mm	0	0	0
TCGA-61-1743	44	20	15	9	0	1329	Dead	No Macroscopic disease	0	0	0
TCGA-61-1895	93	30	35	28	1	44	Alive	No Macroscopic disease	1	0	0
TCGA-61-1899	64	29	19	16	1	256	Alive	No Macroscopic disease	0	0	0
TCGA-61-1900	87	27	35	25	1	176	Alive	No Macroscopic disease	0	0	0
TCGA-61-1901	61	21	25	15	0	347	Dead		1	0	0
TCGA-61-1903	64	24	24	16	1	396	Alive			0	0
TCGA-61-1904	51	25	23	3	0	276	Alive	1-10 mm	0	0	0
TCGA-61-1906	62	23	31	8	0	1038	Dead	11-20 mm	0	0	0
TCGA-61-1907	82	30	39	13	1	952	Alive	11-20 mm	0	0	0
TCGA-61-1910	67	23	26	18	1	1127	Alive	1-10 mm	1	0	0
TCGA-61-1911	41	16	18	7	0	1293	Alive		0	0	0
TCGA-61-1913	65	21	25	19	1	1488	Alive	1-10 mm	1	0	0
TCGA-61-1914	73	30	31	12	1	1722	Alive	>20 mm	0	0	0
TCGA-61-1915	65	24	33	8	1	2061	Alive		0	1	0
TCGA-61-1916						475	Dead	1-10 mm		0	0
TCGA-61-1917	88	31	33	24	1	1321	Dead		0	0	0
TCGA-61-1918	44	27	13	4	0	479	Dead	No Macroscopic disease	0	0	0
TCGA-61-2008-02											
TCGA-61-2017	19	9	6	4	0	1398	Alive	1-10 mm		0	0
TCGA-61-2018	86	32	37	17	1	1381	Alive	No Macroscopic disease		0	0
TCGA-61-2087	63	21	22	20	1	146	Alive	No Macroscopic disease		0	0
TCGA-61-2096	67	27	20	20	1	1776	Alive	No Macroscopic disease		0	0
TCGA-61-2098	68	30	22	16	1	1993	Alive	11-20 mm	0	0	0
TCGA-61-2610											
TCGA-61-2612	61	29	23	9	0	193	Dead	1-10 mm			
TCGA-61-2613	43	18	18	7	0	945	Dead	11-20 mm			
TCGA-61-2614	37	21	12	4	0	262	Dead	1-10 mm			
TCGA-72-4231											
TCGA-72-4232											
TCGA-72-4233											
TCGA-72-4234											
TCGA-72-4235											
TCGA-72-4236											
TCGA-72-4237											
TCGA-72-4238											
TCGA-72-4240											
TCGA-72-4241											
TCGA-OY-A56P	56	21	13	22	0	1207	Alive	1-10 mm			
TCGA-OY-A56Q	39	16	13	10	0	576	Alive	No Macroscopic disease			
TCGA-VG-A8LO	68	20	27	21	1	24	Dead	>20 mm			
TCGA-WR-A838	49	25	21	3	0	304	Dead				

Supplementary Table 2. Annotation of TCGA breast cancer data.

PatientID	HRD score (TAI+LST+LOH)	TAI score	LST score	LOH score	*BRCA1*	*BRCA2*
TCGA-A8-A0	109	33	39	37	0	G-MUT
TCGA-E2-A1	100	29	38	33	G-MUT	0
TCGA-A2-A0	99	34	32	33	0	AMP
TCGA-GI-A2	98	30	38	30	0	0
TCGA-C8-A1	97	29	35	33	HOMDEL	0
TCGA-BH-A1	97	32	37	28	0	0
TCGA-A7-A0	96	38	38	20	0	0
TCGA-AO-A1	95	38	25	32	G-MUT	MUT
TCGA-B6-A4	95	33	32	30	0	0
TCGA-E2-A1	94	37	36	21	0	0
TCGA-A8-A0	93	35	33	25	0	0
TCGA-E2-A1	93	34	34	25	0	0
TCGA-D8-A1	93	33	32	28	0	0
TCGA-AN-A0	92	34	31	27	MUT	0
TCGA-AO-A0	92	25	34	33	0	0
TCGA-AR-A2	91	34	34	23	0	HOMDEL
TCGA-AN-A0	91	29	30	32	0	0
TCGA-BH-A0	89	28	26	35	G-MUT	0
TCGA-A8-A0	88	31	41	16	0	G-MUT
TCGA-A8-A0	88	31	40	17	0	0
TCGA-AR-A0	88	31	30	27	0	0
TCGA-D8-A2	88	29	27	32	0	0
TCGA-E9-A2	87	24	30	33	0	0
TCGA-EW-A1	87	25	31	31	0	0
TCGA-D8-A1	86	27	30	29	0	0
TCGA-GM-A	86	30	37	19	0	0
TCGA-E9-A2	86	27	33	26	0	0
TCGA-AO-A0	86	33	30	23	0	0
TCGA-LL-A4	86	22	31	33	0	0
TCGA-BH-A0	85	29	31	25	MUT	0
TCGA-D8-A1	85	34	28	23	0	0
TCGA-LL-A5	84	28	35	21	MUT	0
TCGA-A2-A0	84	34	33	17	G-MUT	0
TCGA-E9-A2	84	27	32	25	0	0
TCGA-E9-A1	83	28	34	21	MUT	0
TCGA-A2-A3	82	30	33	19	0	0
TCGA-B6-A3	82	19	32	31	0	0
TCGA-BH-A0	81	32	21	28	HOMDEL	0
TCGA-BH-A0	81	31	32	18	0	G-MUT
TCGA-C8-A1	81	23	33	25	0	0
TCGA-EW-A1	81	31	20	30	0	0
TCGA-E2-A1	81	28	35	18	0	0
TCGA-E2-A1	81	31	24	26	0	0
TCGA-AO-A1	80	31	30	19	0	0
TCGA-AO-A1	80	35	23	22	0	0
TCGA-AR-A2	80	33	31	16	0	0
TCGA-A2-A0	80	34	25	21	0	0
TCGA-C8-A2	80	29	26	25	0	0
TCGA-AN-A0	79	26	30	23	G-MUT	0
TCGA-A2-A3	79	26	34	19	0	HOMDEL
TCGA-A2-A0	79	22	37	20	0	G-MUT
TCGA-AR-A0	79	27	29	23	0	0
TCGA-AR-A2	79	20	26	33	0	0
TCGA-EW-A3	79	34	22	23	0	0
TCGA-D8-A1	78	27	31	20	G-MUT	HOMDEL
TCGA-C8-A1	78	27	24	27	0	0
TCGA-E2-A1	78	19	37	22	0	0
TCGA-BH-A1	78	27	32	19	0	G-MUT
TCGA-B6-A0	78	30	22	26	0	0
TCGA-OL-A6	78	26	36	16	0	0
TCGA-OL-A5	77	25	31	21	0	0
TCGA-A2-A0	77	29	30	18	0	0
TCGA-GM-A	77	20	23	34	0	0
TCGA-E2-A1	76	29	29	18	HOMDEL	0
TCGA-A2-A4	76	26	24	26	AMP	0
TCGA-A8-A0	76	30	23	23	0	MUT
TCGA-BH-A0	76	30	19	27	0	0
TCGA-AN-A0	76	26	26	24	0	MUT
TCGA-E9-A2	76	33	29	14	0	0
TCGA-C8-A1	76	29	28	19	0	0
TCGA-BH-A1	76	25	29	22	0	0
TCGA-BH-A0	75	26	23	26	0	0
TCGA-HN-A2	75	28	23	24	0	0
TCGA-A2-A2	74	29	29	16	MUT	0
TCGA-BH-A0	74	20	36	18	0	G-MUT
TCGA-A7-A4	74	22	31	21	0	0
TCGA-AR-A2	73	26	35	12	AMP	HOMDEL
TCGA-D8-A2	73	26	27	20	0	0
TCGA-AO-A0	73	22	28	23	0	0
TCGA-AN-A0	72	23	32	17	G-MUT	0
TCGA-A7-A4	72	29	18	25	0	0
TCGA-D8-A1	72	21	29	22	0	MUT
TCGA-D8-A1	72	17	27	28	0	HOMDEL
TCGA-AR-A2	71	29	20	22	0	0
TCGA-GM-A	71	26	34	11	0	0
TCGA-E2-A1	71	23	28	20	0	0
TCGA-E2-A1	71	27	20	24	0	0
TCGA-E9-A2	71	28	24	19	0	0
TCGA-A8-A0	70	26	22	22	0	0
TCGA-BH-A0	70	20	28	22	0	0
TCGA-BH-A1	70	25	18	27	0	0
TCGA-D8-A1	70	27	16	27	0	0
TCGA-EW-A1	70	30	25	15	0	0
TCGA-A2-A0	69	31	18	20	AMP	0
TCGA-E9-A3	69	27	21	21	0	0
TCGA-E2-A1	69	29	26	14	0	0
TCGA-C8-A1	69	22	29	18	0	0
TCGA-A7-A0	67	24	27	16	0	HOMDEL
TCGA-B6-A0	67	31	14	22	0	0
TCGA-AN-A0	67	30	32	5	0	0
TCGA-BH-A0	67	30	16	21	0	0
TCGA-C8-A1	66	23	27	16	G-MUT	0
TCGA-AN-A0	66	26	27	13	0	HOMDEL
TCGA-A8-A0	66	19	32	15	0	G-MUT
TCGA-E2-A1	66	26	25	15	0	0
TCGA-AC-A3	65	22	24	19	0	0
TCGA-A8-A0	65	25	25	15	0	0
TCGA-A8-A0	65	24	29	12	0	0
TCGA-AO-A0	65	24	28	13	0	0
TCGA-E2-A1	65	20	24	21	0	0
TCGA-A8-A0	65	28	21	16	0	0
TCGA-AQ-A0	65	20	23	22	0	0
TCGA-E2-A1	65	25	19	21	0	0
TCGA-AN-A0	64	21	27	16	G-MUT	0
TCGA-A7-A5	64	28	20	16	0	0
TCGA-A8-A0	64	33	20	11	0	AMP
TCGA-BH-A1	64	25	28	11	0	0
TCGA-AR-A1	64	22	18	24	0	MUT
TCGA-OL-A5	64	23	23	18	0	0
TCGA-BH-A0	64	24	27	13	0	0
TCGA-A8-A0	64	31	17	16	0	0
TCGA-A8-A0	63	22	20	21	0	0
TCGA-BH-A2	63	24	27	12	0	0
TCGA-A2-A4	63	24	21	18	0	0
TCGA-AR-A2	63	19	22	22	0	0
TCGA-A2-A0	63	27	20	16	0	0
TCGA-D8-A1	63	22	27	14	0	0
TCGA-EW-A1	62	16	28	18	0	0
TCGA-BH-A5	62	21	19	22	0	0
TCGA-E9-A5	62	23	18	21	0	0
TCGA-E9-A3	62	17	33	12	0	0
TCGA-BH-A1	62	29	17	16	0	0
TCGA-D8-A2	61	21	22	18	MUT	0
TCGA-A8-A0	61	26	21	14	0	0
TCGA-D8-A1	61	26	29	6	0	HOMDEL
TCGA-E9-A1	61	21	22	18	0	0
TCGA-A7-A0	61	28	17	16	0	0
TCGA-E2-A1	61	23	19	19	0	0
TCGA-E2-A1	61	24	13	24	0	0
TCGA-BH-A0	60	21	22	17	AMP	HOMDEL
TCGA-D8-A1	60	27	18	15	0	0
TCGA-B6-A0	59	24	23	12	0	HOMDEL
TCGA-AR-A0	59	24	19	16	0	0
TCGA-D8-A1	59	27	18	14	0	0
TCGA-E9-A1	59	27	20	12	0	0
TCGA-A2-A0	59	27	16	16	0	0
TCGA-E9-A1	59	25	20	14	0	0
TCGA-BH-A1	58	29	21	8	0	0
TCGA-D8-A1	58	21	28	9	0	0
TCGA-A8-A0	58	22	23	13	0	G-MUT
TCGA-A2-A2	58	19	25	14	0	0
TCGA-AC-A2	58	24	18	16	0	0
TCGA-AR-A2	58	29	13	16	0	0
TCGA-BH-A5	58	21	27	10	0	0
TCGA-A8-A0	57	23	12	22	0	0
TCGA-A2-A0	57	25	17	15	0	0
TCGA-AO-A0	57	23	20	14	0	0
TCGA-E2-A1	57	20	18	19	0	0
TCGA-E9-A1	57	16	27	14	0	0
TCGA-A8-A0	57	25	19	13	0	0
TCGA-EW-A1	57	15	23	19	0	0
TCGA-BH-A0	57	21	27	9	0	0
TCGA-E9-A1	56	23	21	12	0	0
TCGA-BH-A1	56	26	14	16	0	0
TCGA-D8-A1	56	18	23	15	0	0
TCGA-D8-A1	56	17	17	22	0	0
TCGA-A7-A4	56	22	15	19	0	0
TCGA-E2-A1	56	24	16	16	0	0
TCGA-A2-A0	56	19	15	22	0	0
TCGA-D8-A1	55	24	15	16	0	0
TCGA-B6-A4	55	17	12	26	0	0
TCGA-A7-A1	54	23	10	21	AMP	0
TCGA-E9-A1	54	23	19	12	0	0
TCGA-E9-A1	54	21	23	10	0	0
TCGA-JL-A3	54	17	21	16	0	0
TCGA-OL-A5	54	23	7	24	0	0
TCGA-E9-A1	54	25	18	11	0	0
TCGA-GM-A	54	22	23	9	0	0
TCGA-BH-A0	54	23	15	16	0	0
TCGA-C8-A2	54	25	13	16	0	0
TCGA-E9-A1	54	22	15	17	0	0
TCGA-E9-A2	54	23	15	16	0	0
TCGA-A2-A3	54	23	16	15	0	0
TCGA-EW-A1	53	18	25	10	0	0
TCGA-BH-A0	53	20	16	17	0	0
TCGA-A2-A0	53	23	16	14	0	0
TCGA-A2-A0	53	16	16	21	0	0
TCGA-A2-A0	53	23	14	16	0	0
TCGA-A8-A0	53	25	19	9	0	0
TCGA-A7-A2	52	28	17	7	AMP	0
TCGA-B6-A0	52	19	17	16	0	0
TCGA-C8-A1	52	25	20	7	0	0
TCGA-A2-A0	52	26	15	11	0	0
TCGA-EW-A1	52	25	19	8	0	0
TCGA-B6-A0	51	23	17	11	0	0
TCGA-D8-A1	51	18	24	9	0	0
TCGA-AN-A0	51	20	8	23	0	0
TCGA-AR-A1	51	19	12	20	0	0
TCGA-C8-A1	51	22	17	12	0	0
TCGA-E2-A1	51	18	15	18	0	0
TCGA-B6-A0	50	22	19	9	AMP	G-MUT
TCGA-GI-A2	50	22	17	11	0	0
TCGA-A7-A2	50	18	17	15	0	0
TCGA-PE-A5	50	19	17	14	0	HOMDEL
TCGA-A2-A0	50	15	12	23	0	0
TCGA-D8-A1	50	19	12	19	0	0
TCGA-AO-A1	49	21	14	14	MUT	0
TCGA-E2-A1	49	17	11	21	G-MUT	0
TCGA-D8-A2	49	22	10	17	0	0
TCGA-E2-A5	49	21	12	16	0	0
TCGA-B6-A0	49	18	14	17	0	0
TCGA-B6-A0	49	21	12	16	0	0
TCGA-A8-A0	49	28	12	9	0	0
TCGA-AR-A0	49	18	15	16	0	0
TCGA-B6-A0	49	21	11	17	0	0
TCGA-BH-A1	49	16	16	17	0	0
TCGA-EW-A1	49	20	17	12	0	0
TCGA-GM-A	48	23	22	3	0	0
TCGA-AR-A1	48	24	18	6	0	0
TCGA-AN-A0	48	17	15	16	0	0
TCGA-A8-A0	48	20	15	13	0	MUT
TCGA-AR-A1	48	18	12	18	0	0
TCGA-AO-A0	47	23	10	14	0	HOMDEL
TCGA-LL-A5	47	16	20	11	0	0
TCGA-AC-A2	47	19	13	15	0	0
TCGA-E9-A1	47	22	13	12	0	0
TCGA-BH-A0	47	18	14	15	0	G-MUT
TCGA-B6-A1	47	21	12	14	0	0
TCGA-A2-A0	47	16	11	20	0	0
TCGA-A7-A2	47	12	18	17	0	0
TCGA-AO-A0	47	21	17	9	0	0
TCGA-E2-A1	47	20	10	17	0	0
TCGA-E9-A5	47	19	12	16	0	0
TCGA-A1-A0	46	22	18	6	0	0
TCGA-B6-A0	46	19	15	12	0	0
TCGA-A2-A0	46	27	15	4	0	0
TCGA-A8-A0	46	12	23	11	0	0
TCGA-AN-A0	46	19	15	12	0	0
TCGA-A2-A0	46	16	11	19	0	0
TCGA-D8-A1	45	17	19	9	0	0
TCGA-AN-A0	45	23	14	8	0	0
TCGA-C8-A2	45	20	13	12	0	0
TCGA-D8-A1	45	21	15	9	0	0
TCGA-AN-A0	45	17	13	15	0	0
TCGA-AR-A2	45	20	11	14	0	0
TCGA-E2-A5	45	19	11	15	0	0
TCGA-AO-A0	45	17	17	11	0	G-MUT
TCGA-A2-A0	45	17	16	12	0	0
TCGA-AN-A0	45	20	16	9	0	0
TCGA-A7-A2	45	20	8	17	0	0
TCGA-BH-A0	45	16	12	17	0	0
TCGA-AO-A0	45	20	17	8	0	G-MUT
TCGA-AN-A0	45	17	11	17	0	0
TCGA-C8-A1	45	18	15	12	0	0
TCGA-E2-A1	45	15	21	9	0	0
TCGA-A8-A0	44	16	17	11	0	0
TCGA-BH-A1	44	23	11	10	0	0
TCGA-A8-A0	44	15	18	11	0	0
TCGA-EW-A1	44	16	18	10	0	0
TCGA-C8-A1	44	18	10	16	0	0
TCGA-AO-A1	44	21	14	9	0	0
TCGA-B6-A0	44	19	14	11	0	0
TCGA-C8-A1	44	19	16	9	0	0
TCGA-AO-A0	44	17	13	14	0	0
TCGA-BH-A0	44	16	18	10	0	0
TCGA-BH-A0	44	24	10	10	0	0
TCGA-AQ-A5	44	19	4	21	0	0
TCGA-BH-A1	43	18	10	15	HOMDEL	0
TCGA-EW-A1	43	17	7	19	0	0
TCGA-A2-A0	43	19	11	13	0	0
TCGA-BH-A0	43	13	13	17	0	0
TCGA-A2-A0	43	19	9	15	0	0
TCGA-AC-A2	43	17	17	9	0	0
TCGA-AN-A0	43	18	12	13	0	0
TCGA-BH-A0	42	23	12	7	HOMDEL	0
TCGA-AR-A1	42	17	11	14	0	0
TCGA-BH-A2	42	17	10	15	0	0
TCGA-A2-A0	42	18	7	17	0	0
TCGA-BH-A0	42	17	12	13	0	0
TCGA-D8-A1	42	21	9	12	0	0
TCGA-AR-A0	42	17	11	14	0	0
TCGA-AO-A0	41	17	16	8	0	0
TCGA-A2-A2	41	15	19	7	0	0
TCGA-AO-A1	41	18	11	12	0	0
TCGA-B6-A0	41	17	18	6	0	0
TCGA-C8-A1	41	16	14	11	0	0
TCGA-E9-A1	41	14	16	11	0	0
TCGA-E2-A1	41	17	19	5	0	0
TCGA-A1-A0	40	15	16	9	G-MUT	0
TCGA-AN-A0	40	19	12	9	AMP	0
TCGA-AO-A1	40	23	17	0	0	0
TCGA-C8-A2	40	15	12	13	0	0
TCGA-A8-A0	40	18	12	10	0	HOMDEL
TCGA-AC-A6	40	17	12	11	0	0
TCGA-AO-A1	40	16	15	9	0	0
TCGA-BH-A0	40	21	12	7	0	0
TCGA-BH-A1	40	16	16	8	0	0
TCGA-BH-A1	40	17	9	14	0	0
TCGA-C8-A1	40	20	5	15	0	0
TCGA-E9-A2	40	16	17	7	0	0
TCGA-B6-A0	39	17	7	15	0	0
TCGA-AC-A2	39	21	14	4	0	MUT
TCGA-A7-A5	39	18	9	12	0	0
TCGA-BH-A2	39	20	11	8	0	0
TCGA-D8-A1	39	17	10	12	0	0
TCGA-BH-A1	39	10	9	20	0	0
TCGA-E9-A2	39	13	12	14	0	0
TCGA-A2-A0	39	15	14	10	0	0
TCGA-A8-A0	39	17	15	7	0	0
TCGA-B6-A4	39	22	10	7	0	0
TCGA-BH-A0	38	13	8	17	0	0
TCGA-C8-A1	38	19	5	14	0	0
TCGA-A8-A0	38	14	10	14	0	0
TCGA-AO-A0	38	12	12	14	0	0
TCGA-A2-A0	38	12	12	14	0	0
TCGA-A8-A0	38	17	16	5	0	0
TCGA-A2-A0	38	19	14	5	0	0
TCGA-E2-A1	38	16	11	11	0	0
TCGA-C8-A2	38	13	11	14	0	0
TCGA-C8-A1	38	19	6	13	0	0
TCGA-B6-A0	38	16	14	8	0	0
TCGA-BH-A0	38	13	11	14	0	0
TCGA-D8-A1	38	18	12	8	0	0
TCGA-D8-A1	38	18	8	12	0	0
TCGA-E9-A1	38	15	7	16	0	0
TCGA-E9-A1	38	16	5	17	0	0
TCGA-A8-A0	37	15	13	9	0	0
TCGA-A7-A1	37	13	9	15	0	0
TCGA-AN-A0	37	12	8	17	0	0
TCGA-BH-A0	37	12	11	14	0	0
TCGA-A8-A0	37	14	6	17	0	0
TCGA-C8-A1	37	13	10	14	0	0
TCGA-C8-A1	37	13	16	8	0	0
TCGA-D8-A1	37	21	8	8	0	0
TCGA-BH-A4	36	9	12	15	AMP	0
TCGA-BH-A0	36	12	15	9	0	0
TCGA-A8-A0	36	20	10	6	0	0
TCGA-AN-A0	36	15	10	11	0	0
TCGA-A8-A0	36	12	11	13	0	0
TCGA-EW-A1	36	16	6	14	0	0
TCGA-BH-A1	36	11	15	10	0	0
TCGA-D8-A1	36	15	5	16	0	0
TCGA-AR-A2	36	18	10	8	0	0
TCGA-C8-A1	36	20	10	6	0	0
TCGA-EW-A1	36	13	23	0	0	HOMDEL
TCGA-EW-A2	36	11	5	20	0	0
TCGA-D8-A1	36	14	4	18	0	0
TCGA-A2-A0	36	17	10	9	0	0
TCGA-AR-A0	36	14	13	9	0	0
TCGA-BH-A1	36	11	9	16	0	0
TCGA-BH-A1	36	13	9	14	0	0
TCGA-E2-A1	36	18	5	13	0	0
TCGA-EW-A2	36	8	11	17	0	0
TCGA-EW-A2	35	20	8	7	MUT	0
TCGA-A8-A0	35	16	12	7	0	0
TCGA-E2-A1	35	14	12	9	0	0
TCGA-A2-A0	35	15	15	5	0	0
TCGA-A2-A1	35	14	15	6	0	AMP
TCGA-AC-A5	35	18	13	4	0	0
TCGA-AO-A0	35	8	11	16	0	0
TCGA-AR-A1	35	15	7	13	0	0

TCGA-AR-A1	35	11	12	12	0	0
TCGA-D8-A1	35	15	13	7	0	0
TCGA-D8-A2	35	15	13	7	0	0
TCGA-EW-A1	35	14	14	7	0	0
TCGA-A2-A4	34	17	11	6	0	0
TCGA-E2-A1	34	14	10	10	0	0
TCGA-A2-A0	34	15	8	11	0	0
TCGA-C8-A1	34	18	10	6	0	0
TCGA-C8-A1	34	8	18	8	0	0
TCGA-C8-A1	34	13	21	0	0	0
TCGA-A2-A1	34	12	11	11	0	0
TCGA-AC-A5	34	11	6	17	0	0
TCGA-D8-A1	34	16	13	5	0	0
TCGA-A2-A0	33	14	6	13	0	0
TCGA-A8-A0	33	8	8	17	0	0
TCGA-E9-A2	33	10	9	14	0	0
TCGA-B6-A4	33	15	9	9	0	AMP
TCGA-BH-A0	33	14	8	11	0	0
TCGA-AR-A2	33	9	14	10	0	0
TCGA-AO-A1	33	19	8	6	0	AMP
TCGA-AQ-A0	33	14	10	9	0	0
TCGA-AR-A2	33	18	6	9	0	0
TCGA-BH-A1	33	16	10	7	0	0
TCGA-E2-A1	33	11	8	14	0	0
TCGA-A2-A0	32	14	14	4	AMP	0
TCGA-E2-A1	32	11	12	9	0	0
TCGA-A2-A0	32	13	12	7	0	0
TCGA-A8-A0	32	18	7	7	0	0
TCGA-AC-A5	32	7	11	14	0	0
TCGA-A2-A0	32	13	11	8	0	0
TCGA-C8-A2	32	16	11	5	0	0
TCGA-C8-A1	32	9	6	17	0	0
TCGA-E2-A1	32	16	8	8	0	0
TCGA-A8-A0	32	11	7	14	0	0
TCGA-C8-A2	32	12	7	13	0	0
TCGA-E2-A1	32	14	4	14	0	0
TCGA-BH-A1	31	11	11	9	HOMDEL	0
TCGA-A8-A0	31	10	14	7	0	0
TCGA-E2-A1	31	17	7	7	0	0
TCGA-BH-A2	31	11	10	10	0	AMP
TCGA-A8-A0	31	15	5	11	0	0
TCGA-AC-A2	31	13	11	7	0	0
TCGA-BH-A1	31	17	6	8	0	AMP
TCGA-B6-A0	31	15	16	0	0	0
TCGA-AO-A1	31	11	9	11	0	0
TCGA-A2-A1	31	15	6	10	0	0
TCGA-AR-A1	31	10	8	13	0	0
TCGA-AR-A2	31	13	3	15	0	0
TCGA-B6-A0	31	12	4	15	0	0
TCGA-BH-A0	31	14	9	8	0	0
TCGA-A7-A4	31	14	10	7	0	0
TCGA-B6-A0	30	12	8	10	AMP	0
TCGA-E2-A1	30	17	7	6	0	0
TCGA-BH-A0	30	14	8	8	0	0
TCGA-BH-A1	30	13	7	10	0	0
TCGA-B6-A1	30	14	8	8	0	0
TCGA-D8-A1	30	15	10	5	0	0
TCGA-E9-A1	30	11	9	10	0	0
TCGA-A8-A0	30	12	8	10	0	0
TCGA-AR-A2	30	13	8	9	0	0
TCGA-AN-A0	30	15	9	6	0	AMP
TCGA-A8-A0	30	15	12	3	0	0
TCGA-D8-A1	30	8	9	13	0	0
TCGA-E2-A1	30	13	6	11	0	0
TCGA-PE-A5	29	11	11	7	MUT	0
TCGA-B6-A0	29	11	5	13	AMP	0
TCGA-D8-A1	29	9	13	7	0	0
TCGA-BH-A0	29	10	9	10	0	0
TCGA-A8-A0	29	10	10	9	0	0
TCGA-AR-A2	29	7	12	10	0	0
TCGA-A8-A0	29	7	11	11	0	0
TCGA-C8-A2	29	13	9	7	0	0
TCGA-AR-A2	29	13	9	7	0	0
TCGA-B6-A4	29	10	6	13	0	0
TCGA-E9-A1	29	12	12	5	0	0
TCGA-E9-A2	29	13	11	5	0	0
TCGA-A2-A3	29	11	7	11	0	0
TCGA-E2-A1	28	18	6	4	0	0
TCGA-B6-A0	28	7	12	9	0	HOMDEL
TCGA-BH-A0	28	16	6	6	0	0
TCGA-AN-A0	28	12	5	11	0	0
TCGA-A2-A3	28	12	10	6	0	0
TCGA-EW-A1	28	10	10	8	0	0
TCGA-B6-A0	28	11	8	9	0	0
TCGA-B6-A0	28	9	11	8	0	0
TCGA-AO-A0	28	16	8	4	0	0
TCGA-A8-A0	28	11	5	12	0	MUT
TCGA-AO-A0	28	11	9	8	0	0
TCGA-C8-A1	28	10	9	9	0	0
TCGA-A8-A0	28	11	7	10	0	0
TCGA-AO-A0	28	14	10	4	0	0
TCGA-AQ-A1	28	7	11	10	0	0
TCGA-D8-A1	28	10	4	14	0	0
TCGA-E2-A1	28	13	13	2	0	0
TCGA-E2-A1	27	9	4	14	0	0
TCGA-E2-A5	27	12	10	5	0	0
TCGA-AC-A3	27	11	6	10	0	0
TCGA-A2-A0	27	13	7	7	0	0
TCGA-AC-A2	27	11	9	7	0	0
TCGA-A1-A0	27	17	8	2	0	0
TCGA-E9-A2	27	13	5	9	0	0
TCGA-A1-A0	27	8	8	11	0	0
TCGA-A7-A1	27	9	15	3	0	0
TCGA-AN-A0	27	10	11	6	0	0
TCGA-AR-A2	27	8	9	10	0	0
TCGA-B6-A0	27	12	10	5	0	0
TCGA-BH-A0	27	11	5	11	0	0
TCGA-E2-A1	27	10	11	6	0	AMP
TCGA-E9-A2	27	9	10	8	0	0
TCGA-A7-A4	27	11	6	10	0	0
TCGA-EW-A1	26	6	9	11	0	0
TCGA-EW-A4	26	10	7	9	0	0
TCGA-AO-A0	26	7	8	11	0	0
TCGA-AR-A1	26	12	8	6	0	0
TCGA-A2-A3	26	11	10	5	0	0
TCGA-A8-A0	26	10	7	9	0	0
TCGA-AR-A2	26	9	9	8	0	0
TCGA-B6-A0	26	9	5	12	0	0
TCGA-BH-A0	26	12	6	8	0	0
TCGA-D8-A1	26	14	2	10	0	0
TCGA-LL-A5	26	5	9	12	0	0
TCGA-OL-A5	25	9	8	8	0	0
TCGA-E9-A2	25	10	9	6	0	0
TCGA-BH-A1	25	12	8	5	0	0
TCGA-BH-A0	25	7	9	9	0	0
TCGA-BH-A1	25	9	7	9	0	0
TCGA-BH-A1	25	8	8	9	0	0
TCGA-AC-A2	25	12	7	6	0	0
TCGA-A2-A1	25	15	4	6	0	0
TCGA-AQ-A0	25	11	9	5	0	0
TCGA-A1-A0	25	14	3	8	0	0
TCGA-AN-A0	25	16	6	3	0	0
TCGA-AN-A0	25	12	6	7	0	0
TCGA-AQ-A5	25	6	11	8	0	0
TCGA-C8-A1	25	18	3	4	0	0
TCGA-D8-A2	25	10	9	6	0	0
TCGA-E2-A1	25	7	2	16	0	0
TCGA-AC-A2	25	12	6	7	0	0
TCGA-E2-A2	25	14	6	5	0	0
TCGA-A2-A0	24	6	7	11	0	0
TCGA-A8-A0	24	12	6	6	0	0
TCGA-B6-A0	24	9	6	9	0	0
TCGA-B6-A0	24	8	5	11	0	0
TCGA-C8-A1	24	9	7	8	0	0
TCGA-B6-A0	24	5	12	7	0	0
TCGA-EW-A1	24	9	9	6	0	0
TCGA-AR-A1	24	9	5	10	0	0
TCGA-C8-A1	24	8	9	7	0	0
TCGA-A8-A0	24	11	6	7	0	0
TCGA-BH-A0	24	11	4	9	0	0
TCGA-C8-A3	24	9	7	8	0	0
TCGA-E2-A1	24	10	6	8	0	0
TCGA-E9-A2	24	6	8	10	0	0
TCGA-E9-A2	24	10	10	4	0	0
TCGA-GM-A	24	10	6	8	0	0
TCGA-BH-A0	23	10	4	9	0	0
TCGA-BH-A0	23	6	8	9	0	0
TCGA-E2-A1	23	10	6	7	0	0
TCGA-BH-A0	23	10	10	3	0	0
TCGA-E2-A1	23	8	9	6	0	0
TCGA-A1-A0	23	9	7	7	0	0
TCGA-BH-A1	23	15	3	5	0	0
TCGA-D8-A1	23	9	7	7	0	0
TCGA-OL-A5	23	8	8	7	0	0
TCGA-AR-A1	23	9	9	5	0	0
TCGA-C8-A1	22	8	8	6	MUT	MUT
TCGA-A2-A0	22	7	9	6	0	0
TCGA-AC-A5	22	5	13	4	0	0
TCGA-AO-A0	22	9	5	8	0	0
TCGA-C8-A2	22	11	9	2	0	0
TCGA-AR-A0	22	10	5	7	0	0
TCGA-BH-A0	22	10	5	7	0	0
TCGA-A8-A0	22	10	1	11	0	0
TCGA-A8-A0	22	9	6	7	0	0
TCGA-A8-A0	22	9	2	11	0	0
TCGA-LL-A5	22	12	2	8	0	0
TCGA-E9-A5	22	8	4	10	0	0
TCGA-BH-A0	22	11	5	6	0	0
TCGA-A8-A0	22	10	8	4	0	0
TCGA-A8-A0	22	12	5	5	0	0
TCGA-AO-A0	22	9	4	9	0	0
TCGA-BH-A0	22	8	7	7	0	0
TCGA-C8-A1	22	9	3	10	0	0
TCGA-E2-A1	22	13	3	6	0	0
TCGA-E2-A1	22	7	8	7	0	0
TCGA-E2-A1	22	8	6	8	0	0
TCGA-E9-A1	22	10	3	9	0	0
TCGA-A7-A3	22	6	4	12	0	0
TCGA-LQ-A4	22	8	6	8	0	0
TCGA-E9-A1	21	11	7	3	0	0
TCGA-AN-A0	21	9	7	5	0	0
TCGA-A1-A0	21	8	3	10	0	0
TCGA-BH-A2	21	11	4	6	0	0
TCGA-D8-A1	21	5	10	6	0	0
TCGA-B6-A0	21	7	6	8	0	0
TCGA-B6-A0	21	9	7	5	0	0
TCGA-BH-A0	21	12	5	4	0	0
TCGA-BH-A0	21	9	4	8	0	0
TCGA-C8-A2	21	7	8	6	0	0
TCGA-E2-A1	21	11	5	5	0	0
TCGA-E2-A1	21	9	5	7	0	0
TCGA-EW-A1	21	8	8	5	0	0
TCGA-EW-A1	21	7	7	7	0	0
TCGA-JL-A3	21	8	8	5	0	0
TCGA-A8-A0	20	9	6	5	0	0
TCGA-D8-A1	20	7	5	8	0	0
TCGA-AO-A0	20	8	5	7	0	0
TCGA-E2-A1	20	10	4	6	0	0
TCGA-OL-A5	20	11	6	3	0	0
TCGA-A8-A0	20	14	1	5	0	0
TCGA-BH-A0	20	7	10	3	0	0
TCGA-BH-A0	20	9	7	4	0	0
TCGA-A2-A0	20	7	8	5	0	0
TCGA-A2-A0	20	10	8	2	0	0
TCGA-A8-A0	20	9	5	6	0	0
TCGA-AO-A1	20	6	6	8	0	0
TCGA-AR-A2	20	6	5	9	0	0
TCGA-AR-A5	20	7	3	10	0	0
TCGA-BH-A1	20	10	9	1	0	0
TCGA-D8-A1	20	9	2	9	0	0
TCGA-GM-A	20	8	4	8	0	0
TCGA-PE-A5	20	6	9	5	0	0
TCGA-A7-A3	20	5	7	8	0	0
TCGA-BH-A0	19	7	4	8	0	0
TCGA-E9-A1	19	7	8	4	0	0
TCGA-GM-A	19	8	4	7	0	0
TCGA-E2-A1	19	7	6	6	0	0
TCGA-OL-A6	19	8	9	2	0	0
TCGA-A7-A2	19	9	5	5	0	0
TCGA-AC-A2	19	13	1	5	0	0
TCGA-A2-A0	19	8	1	10	0	0
TCGA-A2-A0	19	8	5	6	0	G-MUT
TCGA-D8-A2	19	10	3	6	0	0
TCGA-E2-A2	19	6	5	8	0	0
TCGA-A1-A0	19	9	1	9	0	0
TCGA-A8-A0	19	5	3	11	0	0
TCGA-A8-A0	19	5	7	7	0	0
TCGA-BH-A0	19	7	4	8	0	0
TCGA-D8-A1	19	7	4	8	0	0
TCGA-D8-A1	19	4	9	6	0	0
TCGA-GM-A	19	6	7	6	0	0
TCGA-A1-A0	18	8	2	8	MUT	0
TCGA-EW-A1	18	4	7	7	0	0
TCGA-GM-A	18	7	6	5	0	0
TCGA-A7-A2	18	9	2	7	0	0
TCGA-BH-A0	18	9	5	4	0	0
TCGA-A2-A0	18	11	5	2	0	0
TCGA-A8-A0	18	7	8	3	0	0
TCGA-BH-A0	18	7	1	10	0	0
TCGA-BH-A1	18	6	1	11	0	0
TCGA-D8-A1	18	8	3	7	0	0
TCGA-E2-A1	18	8	9	1	0	0
TCGA-E9-A1	18	10	3	5	0	0
TCGA-E9-A1	18	10	6	2	0	0
TCGA-GM-A	18	7	3	8	0	0
TCGA-B6-A0	17	6	5	6	0	0
TCGA-BH-A1	17	6	7	4	0	0
TCGA-A2-A4	17	4	9	4	0	0
TCGA-A7-A0	17	12	3	2	0	0
TCGA-A2-A0	17	5	9	3	0	0
TCGA-BH-A0	17	5	0	12	0	0
TCGA-BH-A1	17	7	4	6	0	0
TCGA-E9-A1	17	7	3	7	0	0
TCGA-A8-A0	17	5	5	7	0	HOMDEL
TCGA-A8-A0	17	11	1	5	0	0
TCGA-AR-A1	17	10	2	5	0	0
TCGA-AR-A1	17	11	1	5	0	0
TCGA-AR-A5	17	7	1	9	0	0
TCGA-BH-A2	17	5	5	7	0	0
TCGA-C8-A2	17	5	6	6	0	0
TCGA-E9-A1	17	8	6	3	0	0
TCGA-E9-A1	17	6	6	5	0	0
TCGA-EW-A1	17	6	3	8	0	0
TCGA-OL-A5	17	5	8	4	0	0
TCGA-A2-A0	17	6	3	8	0	0
TCGA-E2-A1	17	6	6	5	0	0
TCGA-E9-A5	17	8	1	8	0	0
TCGA-EW-A3	17	7	5	5	0	0
TCGA-A8-A0	16	6	4	6	0	0
TCGA-E2-A1	16	5	4	7	0	0
TCGA-BH-A0	16	6	5	5	0	AMP
TCGA-E2-A1	16	7	5	4	0	0
TCGA-E9-A3	16	10	6	0	0	0
TCGA-A1-A0	16	6	5	5	0	0
TCGA-A2-A0	16	7	4	5	0	0
TCGA-A2-A0	16	6	4	6	0	0
TCGA-A2-A3	16	6	4	6	0	0
TCGA-A7-A0	16	8	4	4	0	0
TCGA-A8-A0	16	8	3	5	0	0
TCGA-A8-A0	16	6	3	7	0	0
TCGA-AO-A1	16	7	5	4	0	0
TCGA-AR-A2	16	4	4	8	0	0
TCGA-BH-A0	16	8	3	5	0	0
TCGA-BH-A0	16	7	5	4	0	0
TCGA-E9-A1	16	6	4	6	0	0
TCGA-A7-A0	16	11	2	3	0	0
TCGA-E2-A1	15	7	4	4	0	0
TCGA-GM-A	15	8	2	5	0	0
TCGA-A2-A2	15	6	3	6	0	0
TCGA-A7-A0	15	7	3	5	0	0
TCGA-A8-A0	15	9	1	5	0	0
TCGA-BH-A1	15	3	7	5	0	0
TCGA-C8-A1	15	4	4	7	0	0
TCGA-D8-A1	15	6	3	6	0	0
TCGA-D8-A1	15	5	3	7	0	0
TCGA-E2-A1	15	7	6	2	0	0
TCGA-E2-A1	15	5	4	6	0	0
TCGA-GM-A	15	3	7	5	0	0
TCGA-A8-A0	14	6	1	7	AMP	0
TCGA-A8-A0	14	6	6	2	0	0
TCGA-AC-A6	14	5	8	1	0	0
TCGA-A2-A2	14	4	6	4	0	0
TCGA-AN-A0	14	8	1	5	0	0
TCGA-A2-A0	14	7	2	5	0	0
TCGA-AO-A0	14	5	1	8	0	0
TCGA-AO-A1	14	6	6	2	0	0
TCGA-AO-A1	14	4	8	2	0	0
TCGA-AR-A1	14	7	6	1	0	0
TCGA-AR-A5	14	4	7	3	0	0
TCGA-B6-A1	14	7	3	4	0	0
TCGA-B6-A1	14	5	7	2	0	0
TCGA-D8-A1	14	5	5	4	0	0
TCGA-D8-A1	14	4	5	5	0	0
TCGA-D8-A1	14	7	2	5	0	AMP
TCGA-D8-A2	14	6	4	4	0	0
TCGA-E2-A1	14	5	4	5	0	0
TCGA-EW-A1	14	4	6	4	0	0
TCGA-A2-A4	14	4	3	7	0	0
TCGA-AC-A2	13	6	6	1	AMP	0
TCGA-A8-A0	13	5	5	3	0	0
TCGA-E2-A1	13	9	1	3	0	AMP
TCGA-AR-A2	13	5	6	2	0	0
TCGA-A7-A1	13	4	4	5	0	0
TCGA-E2-A5	13	5	5	3	0	0
TCGA-EW-A1	13	5	3	5	0	0
TCGA-A2-A0	13	10	0	3	0	0
TCGA-A2-A3	13	5	5	3	0	0
TCGA-A8-A0	13	5	3	5	0	0
TCGA-AC-A2	13	7	3	3	0	0
TCGA-AN-A0	13	5	2	6	0	0
TCGA-AR-A2	13	4	0	9	0	0
TCGA-B6-A0	13	5	4	4	0	0
TCGA-BH-A0	13	6	4	3	0	0
TCGA-BH-A0	13	5	3	5	0	0
TCGA-D8-A2	13	6	2	5	0	0
TCGA-D8-A3	13	6	3	4	0	0
TCGA-E2-A1	13	7	3	3	0	0
TCGA-E2-A1	13	5	5	3	0	0
TCGA-E9-A2	13	6	2	5	0	0
TCGA-AC-A3	13	7	4	2	0	0
TCGA-BH-A1	13	6	3	4	0	0
TCGA-A2-A0	12	7	3	2	AMP	0
TCGA-D8-A2	12	3	2	7	0	MUT
TCGA-A1-A0	12	5	4	3	0	0
TCGA-E2-A1	12	5	3	4	0	0
TCGA-BH-A0	12	8	1	3	0	0
TCGA-BH-A1	12	5	7	0	0	0
TCGA-E2-A1	12	6	3	3	0	0
TCGA-AC-A2	12	5	3	4	0	0
TCGA-AN-A0	12	7	2	3	0	0
TCGA-BH-A2	12	6	2	4	0	0
TCGA-A2-A0	12	5	3	4	0	0
TCGA-A2-A0	12	6	2	4	0	0
TCGA-A2-A0	12	4	3	5	0	0
TCGA-A7-A4	12	5	4	3	0	0
TCGA-A8-A0	12	6	4	2	0	0

TCGA-A8-A0	12	6	2	4	0	0
TCGA-A8-A0	12	6	3	3	0	0
TCGA-AN-A0	12	4	5	3	0	0
TCGA-B6-A2	12	5	4	3	0	0
TCGA-BH-A0	12	5	2	5	0	0
TCGA-BH-A0	12	5	5	2	0	0
TCGA-BH-A1	12	6	1	5	0	0
TCGA-BH-A1	12	5	3	4	0	0
TCGA-BH-A4	12	4	4	4	0	0
TCGA-E2-A1	12	7	1	4	0	0
TCGA-OK-A5	12	5	3	4	0	0
TCGA-AC-A2	12	7	1	4	0	0
TCGA-BH-A1	11	4	4	3	0	0
TCGA-AR-A2	11	4	3	4	0	0
TCGA-BH-A0	11	2	2	7	0	0
TCGA-A2-A1	11	4	4	3	0	0
TCGA-B6-A4	11	3	3	5	0	0
TCGA-AN-A0	11	4	2	5	0	0
TCGA-B6-A4	11	5	2	4	0	0
TCGA-A2-A0	11	4	6	1	0	0
TCGA-A8-A0	11	6	1	4	0	0
TCGA-AN-A0	11	5	3	3	0	0
TCGA-AO-A0	11	4	3	4	0	0
TCGA-AR-A1	11	4	3	4	0	0
TCGA-BH-A0	11	5	2	4	0	0
TCGA-BH-A0	11	5	4	2	0	0
TCGA-C8-A1	11	2	5	4	0	0
TCGA-D8-A1	11	3	4	4	0	0
TCGA-D8-A1	11	2	5	4	0	0
TCGA-E9-A1	11	5	2	4	0	0
TCGA-E9-A1	11	7	3	1	0	0
TCGA-EW-A1	11	6	2	3	0	0
TCGA-EW-A2	11	4	2	5	0	0
TCGA-LL-A5	11	4	4	3	0	0
TCGA-A2-A3	11	4	3	4	0	0
TCGA-A7-A4	11	3	2	6	0	0
TCGA-AC-A2	11	4	3	4	0	0
TCGA-EW-A1	10	3	3	4	HOMDEL	HOMDEL
TCGA-D8-A1	10	5	1	4	0	0
TCGA-A2-A0	10	5	1	4	0	0
TCGA-A2-A0	10	4	3	3	0	0
TCGA-A8-A0	10	2	4	4	0	0
TCGA-AR-A2	10	4	2	4	0	0
TCGA-AR-A5	10	2	5	3	0	0
TCGA-A7-A1	10	4	2	4	0	0
TCGA-E2-A1	10	3	2	5	0	0
TCGA-A2-A0	10	4	3	3	0	0
TCGA-BH-A0	10	3	4	3	0	0
TCGA-A2-A0	10	5	1	4	0	0
TCGA-A2-A0	10	5	2	3	0	0
TCGA-A2-A1	10	4	4	2	0	0
TCGA-A8-A0	10	3	4	3	0	0
TCGA-A8-A0	10	5	3	2	0	0
TCGA-AC-A3	10	5	2	3	0	0
TCGA-AQ-A1	10	5	2	3	0	0
TCGA-AR-A2	10	5	1	4	0	0
TCGA-BH-A0	10	2	4	4	0	0
TCGA-BH-A0	10	5	0	5	0	0
TCGA-D8-A1	10	5	2	3	0	0
TCGA-A7-A3	10	7	2	1	0	0
TCGA-AC-A3	10	4	2	4	0	0
TCGA-AC-A3	10	4	2	4	0	0
TCGA-GM-A	10	5	2	3	0	0
TCGA-AN-A0	9	3	3	3	0	0
TCGA-A2-A2	9	4	2	3	0	0
TCGA-BH-A0	9	6	1	2	0	0
TCGA-A7-A0	9	7	0	2	0	0
TCGA-AN-A0	9	4	2	3	0	0
TCGA-AR-A2	9	3	3	3	0	0
TCGA-BH-A0	9	5	2	2	0	0
TCGA-BH-A0	9	4	1	4	0	0
TCGA-BH-A0	9	3	2	4	0	0
TCGA-BH-A0	9	4	1	4	0	0
TCGA-C8-A1	9	5	1	3	0	0
TCGA-D8-A1	9	3	2	4	0	0
TCGA-E2-A1	9	4	4	1	0	0
TCGA-EW-A1	9	6	1	2	0	0
TCGA-GM-A	9	3	3	3	0	0
TCGA-HN-A	9	6	2	1	0	0
TCGA-OL-A6	9	4	1	4	0	0
TCGA-A7-A3	9	0	5	4	0	0
TCGA-AC-A3	9	4	1	4	0	0
TCGA-A2-A0	8	5	1	2	0	0
TCGA-E9-A1	8	2	4	2	0	0
TCGA-D8-A1	8	3	2	3	0	0
TCGA-OL-A5	8	5	2	1	0	0
TCGA-EW-A1	8	5	1	2	0	MUT
TCGA-A2-A0	8	3	2	3	0	0
TCGA-A2-A0	8	4	1	3	0	0
TCGA-A2-A3	8	3	1	4	0	0
TCGA-A8-A0	8	3	2	3	0	0
TCGA-A8-A0	8	5	1	2	0	0
TCGA-AC-A3	8	3	3	2	0	0
TCGA-AR-A2	8	5	1	2	0	0
TCGA-BH-A0	8	4	1	3	0	0
TCGA-E2-A1	8	5	0	3	0	0
TCGA-E2-A1	8	3	3	2	0	0
TCGA-E9-A2	8	5	0	3	0	0
TCGA-GM-A	8	5	0	3	0	0
TCGA-AC-A2	8	3	4	1	0	0
TCGA-D8-A3	8	5	1	2	0	0
TCGA-AN-A0	7	3	2	2	0	MUT
TCGA-AR-A2	7	4	0	3	0	0
TCGA-A2-A0	7	3	0	4	0	0
TCGA-B6-A0	7	3	1	3	0	0
TCGA-A2-A0	7	4	1	2	0	0
TCGA-A7-A5	7	2	3	2	0	0
TCGA-A8-A0	7	2	2	3	0	0
TCGA-AR-A1	7	4	0	3	0	0
TCGA-AR-A2	7	4	0	3	0	0
TCGA-AR-A2	7	4	0	3	0	0
TCGA-B6-A0	7	3	3	1	0	0
TCGA-B6-A0	7	3	1	3	0	0
TCGA-BH-A0	7	3	2	2	0	0
TCGA-BH-A0	7	2	1	4	0	0
TCGA-BH-A2	7	3	2	2	0	0
TCGA-C8-A3	7	3	3	1	0	0
TCGA-D8-A1	7	2	2	3	0	0
TCGA-E2-A1	7	4	1	2	0	0
TCGA-GM-A	7	3	1	3	0	0
TCGA-AC-A3	7	3	0	4	0	0
TCGA-E2-A3	7	2	2	3	0	0
TCGA-A8-A0	6	3	1	2	0	HOMDEL
TCGA-A2-A0	6	3	1	2	0	0
TCGA-B6-A0	6	2	2	2	0	0
TCGA-BH-A1	6	1	4	1	0	0
TCGA-A7-A0	6	3	1	2	0	0
TCGA-E2-A1	6	4	1	1	0	0
TCGA-BH-A1	6	4	0	2	0	0
TCGA-B6-A0	6	2	1	3	0	0
TCGA-A2-A0	6	3	0	3	0	0
TCGA-A2-A0	6	3	1	2	0	0
TCGA-A7-A2	6	4	0	2	0	0
TCGA-AO-A1	6	2	3	1	0	0
TCGA-B6-A0	6	3	1	2	0	0
TCGA-B6-A0	6	3	1	2	0	0
TCGA-B6-A0	6	3	1	2	0	0
TCGA-BH-A0	6	3	0	3	0	0
TCGA-BH-A0	6	3	1	2	0	0
TCGA-BH-A0	6	4	0	2	0	0
TCGA-BH-A0	6	3	0	3	0	0
TCGA-BH-A0	6	4	0	2	0	0
TCGA-BH-A1	6	4	1	1	0	0
TCGA-D8-A1	6	4	0	2	0	0
TCGA-OL-A5	6	4	1	1	0	0
TCGA-E9-A5	6	2	3	1	0	0
TCGA-E2-A1	5	2	1	2	MUT	0
TCGA-A7-A5	5	2	1	2	0	0
TCGA-AC-A3	5	4	1	0	0	0
TCGA-BH-A0	5	2	1	2	0	0
TCGA-AO-A0	5	2	2	1	0	0
TCGA-D8-A1	5	2	2	1	0	0
TCGA-AC-A2	5	3	1	1	0	0
TCGA-A2-A1	5	3	1	1	0	0
TCGA-E2-A1	5	3	0	2	0	0
TCGA-A8-A0	5	1	1	3	0	0
TCGA-AO-A1	5	3	1	1	0	0
TCGA-B6-A0	5	3	0	2	0	0
TCGA-BH-A0	5	2	3	0	0	0
TCGA-BH-A0	5	3	0	2	0	0
TCGA-BH-A0	5	3	0	2	0	0
TCGA-BH-A1	5	3	0	2	0	0
TCGA-D8-A2	5	3	0	2	0	0
TCGA-D8-A2	5	2	2	1	0	0
TCGA-E2-A1	5	3	0	2	0	0
TCGA-E2-A1	5	3	1	1	0	0
TCGA-A8-A0	5	3	0	2	0	0
TCGA-GM-A	4	2	0	2	0	0
TCGA-AC-A3	4	0	4	0	0	0
TCGA-LL-A5	4	1	3	0	0	0
TCGA-E9-A2	4	2	0	2	0	0
TCGA-OL-A5	4	3	0	1	0	0
TCGA-BH-A0	4	2	2	0	0	0
TCGA-D8-A1	4	3	1	0	0	0
TCGA-D8-A2	4	3	1	0	0	0
TCGA-E2-A1	4	3	0	1	0	0
TCGA-E2-A1	4	3	0	1	0	0
TCGA-E9-A1	4	0	3	1	0	0
TCGA-E9-A5	3	2	1	0	0	0
TCGA-AN-A0	3	1	1	1	0	0
TCGA-D8-A2	3	0	2	1	0	0
TCGA-AN-A0	3	1	0	2	0	0
TCGA-AO-A0	3	2	0	1	0	0
TCGA-D8-A1	3	3	0	0	0	0
TCGA-GM-A	3	1	2	0	0	0
TCGA-OL-A5	2	0	2	0	0	0
TCGA-E9-A1	2	1	1	0	0	0
TCGA-AC-A3	2	1	1	0	0	0
TCGA-GM-A	2	1	1	0	0	0
TCGA-E9-A3	2	1	1	0	0	0
TCGA-E2-A1	1	0	1	0	0	0
TCGA-BH-A1	1	0	1	0	0	0
TCGA-A2-A0	1	0	1	0	0	0
TCGA-A2-A0	1	1	0	0	0	0
TCGA-B6-A0	1	0	1	0	0	0
TCGA-D8-A1	1	1	0	0	0	0
TCGA-E9-A2	1	1	0	0	0	0
TCGA-LL-A4	1	0	1	0	0	0
TCGA-A2-A4	0	0	0	0	MUT	0
TCGA-A8-A0	0	0	0	0	0	0
TCGA-A1-A0	0	0	0	0	0	0
TCGA-A2-A0	0	0	0	0	0	0
TCGA-A8-A0	0	0	0	0	0	0
TCGA-AO-A0	0	0	0	0	0	0
TCGA-EW-A1	0	0	0	0	0	0
TCGA-BH-A2	0	0	0	0	0	0
TCGA-AR-A1	0	0	0	0	0	0
TCGA-E2-A1	0	0	0	0	0	0
TCGA-OL-A6	0	0	0	0	0	0
TCGA-A2-A1	0	0	0	0	0	0
TCGA-C8-A1	0	0	0	0	0	0
TCGA-A2-A2	0	0	0	0	0	MUT
TCGA-A2-A3	0	0	0	0	0	0
TCGA-A7-A3	0	0	0	0	0	0
TCGA-AC-A2	0	0	0	0	0	0
TCGA-AO-A0	0	0	0	0	0	0
TCGA-AO-A1	0	0	0	0	0	0
TCGA-AR-A2	0	0	0	0	0	0
TCGA-AR-A2	0	0	0	0	0	0
TCGA-BH-A0	0	0	0	0	0	0
TCGA-BH-A4	0	0	0	0	0	0
TCGA-D8-A4	0	0	0	0	0	0
TCGA-E2-A1	0	0	0	0	0	0
TCGA-E9-A2	0	0	0	0	0	0
TCGA-EW-A1	0	0	0	0	0	0
TCGA-A2-A4	0	0	0	0	0	0
TCGA-E2-A1	0	0	0	0	0	0
TCGA-GM-A	0	0	0	0	0	0
TCGA-A8-A0	105	36	40	29		
TCGA-AR-A0	105	31	35	39		
TCGA-AR-A0	94	31	34	29		
TCGA-A1-A0	93	34	30	29		
TCGA-LL-A8	93	37	29	27		
TCGA-S3-AA	88	23	31	34		
TCGA-B6-A0	87	28	31	28		
TCGA-A7-A6	85	22	33	30		
TCGA-AC-A8	84	28	30	26		
TCGA-AO-A1	83	30	31	22		
TCGA-B6-A0	80	30	33	17		
TCGA-B6-A0	79	29	33	17		
TCGA-A7-A6	76	29	34	13		
TCGA-AR-A0	73	23	32	18		
TCGA-AC-A6	71	25	16	30		
TCGA-PL-A8	71	29	26	16		
TCGA-S3-AA	67	24	30	13		
TCGA-OL-A6	67	29	26	12		
TCGA-LL-A6	66	25	20	21		
TCGA-AR-A0	66	26	26	14		
TCGA-A7-A6	65	28	9	28		
TCGA-PL-A8	64	23	19	22		
TCGA-AR-A0	61	25	21	15		
TCGA-UU-A	60	23	21	16		
TCGA-LL-A7	60	22	17	21		
TCGA-C8-A8	54	17	21	16		
TCGA-EW-A6	52	23	10	19		
TCGA-A7-A6	49	18	18	13		
TCGA-AN-A0	49	24	8	17		
TCGA-LL-A7	49	19	14	16		
TCGA-E9-A1	47	19	10	18		
TCGA-EW-A6	46	21	8	17		
TCGA-LL-A7	44	22	8	14		
TCGA-EW-A1	43	19	12	12		
TCGA-S3-A6	42	20	13	9		
TCGA-UL-A	41	17	21	3		
TCGA-AR-A0	39	15	14	10		
TCGA-BH-A8	38	9	21	8		
TCGA-EW-A6	38	15	10	13		
TCGA-3C-AA	37	17	13	7		
TCGA-C8-A9	37	9	13	15		
TCGA-LD-A7	36	12	11	13		
TCGA-BH-A0	36	17	11	8		
TCGA-OL-A6	34	10	19	5		
TCGA-3C-AA	34	16	11	7		
TCGA-S3-AA	34	14	8	12		
TCGA-BH-A0	33	14	11	8		
TCGA-AR-A0	33	12	8	13		
TCGA-B6-A0	33	10	10	13		
TCGA-WT-A	32	13	5	14		
TCGA-E2-A1	32	12	7	13		
TCGA-AC-A8	31	13	13	5		
TCGA-AC-A6	31	17	8	6		
TCGA-BH-A6	30	7	10	13		
TCGA-EW-A6	30	9	17	4		
TCGA-E2-A5	28	9	4	15		
TCGA-AC-A7	28	9	11	8		
TCGA-S3-A6	28	13	9	6		
TCGA-AC-A6	27	8	5	14		
TCGA-C8-A8	27	8	6	13		
TCGA-LL-A7	26	12	8	6		
TCGA-BH-A0	24	8	7	9		
TCGA-XX-A8	24	8	4	12		
TCGA-5T-A9	23	8	6	9		
TCGA-AR-A0	23	9	5	9		
TCGA-Z7-A8	23	9	5	9		
TCGA-EW-A1	23	10	9	4		
TCGA-AC-A6	22	9	7	6		
TCGA-BH-A0	22	5	9	8		
TCGA-S3-AA	22	6	8	8		
TCGA-S3-AA	22	7	7	8		
TCGA-AQ-A7	21	8	6	7		
TCGA-A7-A4	20	9	3	8		
TCGA-LL-A6	20	7	9	4		
TCGA-3C-AA	19	6	6	7		
TCGA-AO-A0	18	5	3	10		
TCGA-AC-A4	18	8	2	8		
TCGA-C8-A8	18	10	8	0		
TCGA-D8-A7	18	5	4	9		
TCGA-EW-A6	18	8	5	5		
TCGA-4H-A	17	7	6	4		
TCGA-LD-A7	17	6	5	6		
TCGA-LL-A9	17	8	2	7		
TCGA-D8-A1	17	9	4	4		
TCGA-BH-A6	16	6	3	7		
TCGA-LD-A6	16	6	4	6		
TCGA-OL-A6	16	4	8	4		
TCGA-S3-A6	16	6	6	4		
TCGA-W8-A8	16	7	5	4		
TCGA-WT-A	16	7	4	5		
TCGA-AO-A1	16	5	7	4		
TCGA-D8-A7	15	5	3	7		
TCGA-XX-A8	15	6	4	5		
TCGA-AC-A8	14	6	3	5		
TCGA-LD-A7	14	6	3	5		
TCGA-AC-A3	13	7	1	5		
TCGA-AC-A7	13	4	9	0		
TCGA-BH-A	13	4	4	5		
TCGA-OL-A6	13	6	2	5		
TCGA-BH-A8	12	5	4	3		
TCGA-E9-A6	12	5	2	5		
TCGA-V7-A7	11	5	1	5		
TCGA-OL-A6	11	6	0	5		
TCGA-Z7-A8	11	3	8	0		
TCGA-OL-A9	10	3	5	2		
TCGA-PL-A8	10	6	2	2		
TCGA-LL-A7	10	3	3	4		
TCGA-5L-AA	10	3	3	4		
TCGA-AR-A0	10	4	6	0		
TCGA-BH-A0	10	5	5	0		
TCGA-5L-AA	9	3	3	3		
TCGA-BH-A0	9	5	2	2		
TCGA-D8-A7	9	5	2	2		
TCGA-3C-AA	8	3	2	3		
TCGA-OL-A6	8	4	2	2		
TCGA-BH-A1	8	4	4	0		
TCGA-B6-A0	7	4	1	2		
TCGA-OL-A6	7	4	1	2		
TCGA-E2-A5	6	1	0	5		
TCGA-LL-A6	6	3	1	2		
TCGA-S3-AA	5	1	3	1		
TCGA-AC-A6	5	3	0	2		
TCGA-AC-A8	5	2	0	3		
TCGA-BH-A8	4	2	1	1		
TCGA-A2-A0	3	2	1	0		
TCGA-PL-A8	3	0	3	0		
TCGA-S3-AA	1	1	0	0		
TCGA-A2-A0	1	0	1	0		
TCGA-LD-A9	0	0	0	0		
TCGA-AR-A250					0	0
TCGA-AN-A0FT					0	0
TCGA-A1-A0SQ					0	0
TCGA-AN-A0FZ					0	0
TCGA-BH-A18P					0	0
TCGA-AO-A0JL					HOMDEL	0
TCGA-AQ-A04L					0	0
TCGA-MS-A51U					0	0
TCGA-AR-A1AT						

TCGA-E2-A1LS
TCGA-E2-A9RU
TCGA-A8-A07S
TCGA-EW-A424
TCGA-FA-A7DS
TCGA-LL-A442
